# Patterns of polymorphism and selection in the subgenomes of the allopolyploid *Arabidopsis kamchatica*

**DOI:** 10.1038/s41467-018-06108-1

**Published:** 2018-09-25

**Authors:** Timothy Paape, Roman V. Briskine, Gwyneth Halstead-Nussloch, Heidi E. L. Lischer, Rie Shimizu-Inatsugi, Masaomi Hatakeyama, Kenta Tanaka, Tomoaki Nishiyama, Renat Sabirov, Jun Sese, Kentaro K. Shimizu

**Affiliations:** 1 Department of Evolutionary Biology and Environmental Studies, University of Zurich, Winterthurerstrasse 190, CH-8057 Zurich, Switzerland; 2 Department of Plant and Microbial Biology, University of Zurich, Zollikerstrasse 107, CH-8008 Zurich, Switzerland; 3 Department of Environmental Systems Science, ETH Zurich, CH-8092 Zurich, Switzerland; 4 Swiss Institute of Bioinformatics (SIB), Lausanne, 1015 Switzerland; 5 Functional Genomics Center Zurich, Winterthurerstrasse 190, CH-8057 Zurich, Switzerland; 6 Sugadaira Montane Research Center, University of Tsukuba, Nagano Ueda, 386-2204 Japan; 7 Advanced Science Research Center, Kanazawa University, 13-1 Takara-machi, Kanazawa, 920-0934 Japan; 8 Institute of Marine Geology and Geophysics, Far East Branch, Russian Academy of Sciences, Nauki street, 1-B, Yuzhno-Sakhalinsk, 693022 Russian Federation; 9 Artificial Intelligence Research Center, National Institute of Advanced Industrial Science and Technology (AIST), Tokyo, 135-0064 Japan; 10 AIST-Tokyo Tech Real World Big-Data Computation Open Innovation Laboratory, Tokyo, 152-8550 Japan; 11 Kihara Institute for Biological Research, Yokohama City University, 641-12 Maioka, Yokohama, 244-0813 Japan

## Abstract

Genome duplication is widespread in wild and crop plants. However, little is known about genome-wide selection in polyploids due to the complexity of duplicated genomes. In polyploids, the patterns of purifying selection and adaptive substitutions may be affected by masking owing to duplicated genes or homeologs as well as effective population size. Here, we resequence 25 accessions of the allotetraploid *Arabidopsis kamchatica*, which is derived from the diploid species *A. halleri* and *A. lyrata*. We observe a reduction in purifying selection compared with the parental species. Interestingly, proportions of adaptive non-synonymous substitutions are significantly positive in contrast to most plant species. A recurrent pattern observed in both frequency and divergence–diversity neutrality tests is that the genome-wide distributions of both subgenomes are similar, but the correlation between homeologous pairs is low. This may increase the opportunity of different evolutionary trajectories such as in the *HMA4* gene involved in heavy metal hyperaccumulation.

## Abstract (web summary)

Despite the prevalence of genome duplication in plants, little is known about the evolutionary patterns of entire subgenomes. Here the authors resequence allopolyploid *Arabidopsis kamchatica* genome to estimate diversity, linkage disequilibrium and strengths of both positive and purifying selection.

## Introduction

Genome duplication is a widespread evolutionary force in plants. As many as 35% of vascular plants are recent polyploid species^1^ and increased ploidy is particularly common in crops^2^. The abundance of polyploid species in plants motivated speculation and theoretical analysis on the advantages and disadvantages of genome duplication^3,4^. However, compared with diploid species, much less is known about the genome-wide patterns of polymorphism and selection in polyploids due to genome complexity^5^. Major difficulties in genome-scale analyses result from the large genome sizes of polyploids and the high similarity between the duplicated chromosomes. Recent advances in next-generation sequencing and bioinformatic tools^6,7^ are enabling the use of genome-wide data to study polymorphisms and transcriptomics patterns for entire subgenomes in newly emerging model polyploids^8–11^.

Genome-wide strengths of positive and purifying selection can be quantified using several complementary approaches. Frequency-based tests using site-frequency spectra (SFS) such as Tajima’s *D* and Fay and Wu’s *H* statistics can detect rare or common polymorphisms that are due to purifying or positive selection. Divergence–diversity-based tests compare interspecific divergence (from an outgroup) with intraspecific polymorphism to identify positive selection on amino-acid substitutions^12^. These tests include several derivatives of the McDonald–Kreitman test^13^ (or MK tests), such as the DoS neutrality index^14^, and methods to estimate the distribution of fitness effects (DFEs) and proportion of adaptive substitutions (*α*)^13^ in genome-wide data. Theoretical and empirical studies in plant species using these methods^15,16^ showed that the strengths of selection are affected by species–specific characteristics, such as the effective population size (*N*_e_), mating system, and genome duplication, which are mutually interacting. In particular, species with low *N*_e_ typically have the highest proportions of neutral mutations^15,17^, while in species with a large *N*_e_, there is greater efficacy of selection on deleterious or adaptive substitutions^8,18–20^.

Allopolyploidization should have a profound effect on patterns of polymorphism and selection. First, the redundancy of duplicated gene copies of similar function from different parents (homeologs) may affect the strength of selection. At the early stages, genome duplication may increase evolutionary rates of duplicated genes^21,22^ and may facilitate the evolution of a new adaptive function because the original function can be retained in other copies (so-called neofunctionalization model)^23,24^. In contrast, the additional copy may mask the effect of adaptive and deleterious substitutions^4,16^. Second, polyploidization must involve a reduction in *N*_e_ due to a bottleneck during speciation. In addition, polyploid speciation is typically associated with the transition from outcrossing to self-fertilization, which reduces *N*_e_ by at least a half compared with the parental species^25^. While studies of selection in polyploids are very limited, a recent empirical study in the allotetraploid *Capsella bursa-pastoris* analyzed about 40% of the genome and showed a decrease in the efficacy of purifying selection in one of the subgenomes but an increase in another subgenome^8^. Further empirical studies are necessary to compare the consequences of genome duplication in polyploid species.

The genus *Arabidopsis* has both auto- and allopolyploid species in addition to the more well-studied diploid relatives^26^. *Arabidopsis kamchatica*^27^ is a recent allopolyploid (estimated 20,000–250,000 years ago)^28^ derived from the two diploid species *A. halleri* (particularly subsp. *gemmifera* distributed in East Asia) and *A. lyrata* (particularly subsp. *petraea* from Far East Russia)^29–31^. The two diploid parents are predominantly self-incompatible (SI) while a transition to selfing accompanied allopolyploid formation^28^. The genome size (about 450 Mb) is relatively small among polyploid species^32,33^, which is an advantage for resequencing. The species distribution of *A. kamchatica* is very broad, ranging from Taiwan, Japan, Far East Russia, Alaska and the Pacific Northwest, USA. The high variation in latitude and altitude compared with the parental species^34,35^ suggests that merging the diploid transcriptional networks and parental adaptations provided the allopolyploid with plasticity to inhabit diverse environments^10^.

To understand the ecological distributions of polyploids, genetically tractable traits are essential. Heavy metal tolerance and hyperaccumulation likely influenced ecological divergence and speciation between the parental species of *A. kamchatica* (*A. halleri* and *A. lyrata*) due to adaptive mutations in metal transporter genes such as *HEAVY METAL ATPASE4* (*HMA4*)^36,37^. The *HMA4* locus has been shown to be the primary transporter of cadmium and zinc from roots to shoots in *A. halleri* because of a tandem triplication and enhanced *cis*-regulation^37^, while only a single copy of *HMA4* exists in the non-hyperaccumulators *A. lyrata* and *A. thaliana*^38^. *A. kamchatica* inherited hyperaccumulation from the diploid parent *A. halleri*, although attenuated expression of *halleri-*derived *HMA4* and putatively inhibiting *lyrata*-derived factors reduced the trait to about half of that in *A. halleri*^10^. Estimates of genetic diversity surrounding the *HMA4* region in *A. halleri* suggest a hard selective sweep^39^, which may have predated the formation of *A. kamchatica*^10^.

Here, we use de novo assemblies of the closest diploid relatives of *A. kamchatica* to sort Illumina reads to their respective subgenomes using a distribution-wide collection of 25 natural allopolyploid accessions and analyze the data in combination with published data of the two parental species. We use population genomics to ask: (a) What is the level of genome-wide diversity compared with diploid outcrossing and selfing *Arabidopsis* species? (b) Are there differences in polymorphism, allele frequencies, linkage disequilibrium (LD), and selection between subgenomes? (c) Do pairs of homeologs tend to show similar patterns in diversity and neutrality? (d) Does the *HMA4* locus show significant differences in genetic diversity between homeologs and how does diversity surrounding this locus compare with the genome-wide average? (e) What proportions of the subgenomes show neutral, deleterious, or adaptive mutations and how do they differ from the diploid parents? and (f) Are there high frequencies of loss-of-function mutations in either subgenome? Together, these plant accessions and polymorphism data will serve as a core diversity panel for further studies of genotype-phenotype associations and the genetic architecture of complex traits using larger collections of globally collected samples.

## Results

### Reference genome assembly and allopolyploid resequencing

To sort Illumina reads of *A. kamchatica* to their parentally derived subgenomes, we generated long mate-pair de novo assemblies of *A. lyrata* subsp. *petraea* (also called *A. petraea* subsp. *umbrosa*) in addition to East Asian *A. halleri* subsp. *gemmifera*, which we previously reported^38^. Assembly statistics indicated that the *A. lyrata* and *A. halleri* reference genomes have scaffold N50 of 1.2 and 0.7 Mb, comprising 1675 and 2239 scaffolds, respectively (Table 1, Supplementary Tables 1 and 2 for gene annotation statistics, Supplementary Note 1), providing opportunities to compare diversity over very large syntenic regions in the allopolyploid subgenomes.

**Table 1:** Reference genome assembly statistics

Assembly statistics	*A. lyrata* ^a^	*A. halleri* ^b^
Length (bp)	175,182,717	196,243,198
Missing (%)^c^	12.75	14.81
Scaffolds	1675	2239
Shortest scaffolds (bp)	940	932
Longest scaffolds (bp)	6,771,235	4,302,264
Scaffold N50 length (bp)	1,260,070	712,249
Scaffold N50 count	38	71
NG50 length (bp)	804,357	489,153
NG50 count	63	117
Genome size (FC)^d^	225 Mb	250 Mb

^a^v2.2 of Siberian *A. lyrata* subsp. *petraea* assembled in this study

^b^*A*. *halleri* assembly previously reported in Briskine et al.^38^

^c^Missing data counted as number of Ns in the assembly and are a percentage of total length

^d^Genome size measured by flow cytometry (FC)

We sorted reads of 25 individuals from a distribution-wide collection (Supplementary Fig. 1 and Supplementary Table 3) of *A. kamchatica* to their parental origins by first aligning each read to both parental genomes and then classified the reads as origin reads (*halleri*-derived = H-origin, *lyrata*-derived = L-origin) using algorithms that quantify mismatches to either parent^32^. Our accessions had on average 12.5× coverage for the H-origin subgenome (range 5.2–20.7×) and on average 10.7× coverage for the L-origin subgenome (range 4.3–17.7×). Previously, pyrosequencing was used in two studies to detect ratios of parentally derived SNPs to validate homeolog-specific expression (RNA-seq) in 10 genes^10,32^ where the same read sorting pipeline was used. Here we used homeolog-specific PCR and Sanger sequencing to validate SNPs and read sorting for 12 genes. We showed that reads were accurately assigned to their respective subgenomes (Supplementary Note 2). The alignments consisted of 285 divergent positions between the two homeologs. Because most of these positions were represented by two homeologs of multiple (up to three) *A. kamchatica* individuals, we counted a total of 1375 SNP sites. Among these, only three SNPs (0.2%) in Sanger sequences were different from the NGS data, validating the read sorting and SNP calling. After filtering for SNP quality and coverage, our resequencing dataset resulted in 1,674,191 H*-*origin and 1,930,341 L-origin SNPs. Using the parental genome assemblies, we identified 23,529 homeologous coding sequences using reciprocal best BLAST (best-to-best) hits (Supplementary Table 2) representing 72% of the annotated genes. Among these, 21,433 (H-origin) and 21,472 (L-origin) showed orthology to *A. thaliana*, representing ~67% of the annotated genes in both subgenomes.

### Genome-wide nucleotide diversity in *A. kamchatica*

**Fig. 1:**
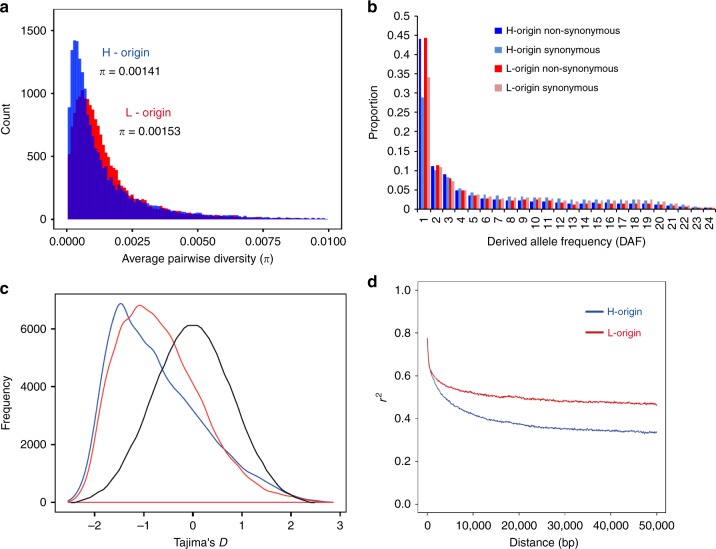
Genome-wide diversity and linkage disequilibrium. **a** Nucleotide diversity (average pairwise diversity, *π*) of *halleri*-origin (H-origin) and *lyrata-*origin (L-origin) coding sequences. **b** H-origin and L-origin genes show no significant differences in proportions of non-synonymous and synonymous substitutions (*χ*^2^-test, *p*-value = 0.58), and the majority of substitutions are at low frequency. **c** Tajima’s *D* distributions for both genomes (blue density curve = H-origin, red density curve = L-origin) show departures from neutrality (black density curve where neutral = 0); mean values for both distributions are negative (Table 1). **d** The mean decay of linkage disequilibrium (LD) estimated using 50 kb sliding windows shows mean LD decay <20 kb for both H-origin (blue) and L-origin (red) genomes

We examined the patterns of nucleotide diversity for ca. 21,000 coding sequences of both *halleri-* and *lyrata*-derived homeologs in *A. kamchatica* that could be aligned to *A. thaliana* orthologs as the outgroup. We found that both subgenomes showed similar mean values of nucleotide diversity (*π*) (*π*_coding_ = 0.0014 bp^–1^ for the *halleri* subgenome, *π*_coding_ = 0.0015 bp^–1^ for the *lyrata* subgenome, and *π*_coding_ = 0.0015 bp^–1^ when combined), although the *lyrata*-derived homeologs showed slightly broader ranges in *π* (Table 2, Fig. 1a). Nucleotide diversity at synonymous sites (*π*_syn_) was also similar for the two subgenomes with a slightly higher value for the *lyrata* subgenome (*π*_syn_ = 0.0049) than the *halleri* subgenome (*π*_syn_ = 0.0044). Sliding window analysis including non-coding regions also showed comparable values (Supplementary Table 4). The nucleotide diversity in *A. kamchatica* is about six times lower than European *A. halleri* and *A. lyrata* (*π*_syn_ = 0.028 for *A. halleri* and 0.028 for *A. lyrata*) estimated using resequencing data reported by Novikova et al.^31^ (Supplementary Tables 5 and 6) and is more similar to that of *A. thaliana* (*π*_syn_ = 0.0059–0.007)^17,31,40^. The ancestor of the genus *Arabidopsis* must have been a SI diploid species like present-day *A. lyrata* and *A. halleri*^25^, suggesting similar reductions in genetic diversity in the lineages of *A. kamchatica* and *A. thaliana*.

**Table 2:** Diversity statistics for coding sequences of *A. kamchatica* homeologs^a^

	*halleri* homeologs			*lyrata* homeologs				Combined		
Statistic	Mean	sd	*n*	Mean	sd	*n*	*r*	Mean	sd	*n*
*π*_total_	0.0014	0.0019	21,419	0.0015	0.0018	21,463	0.27	0.0015	0.0015	20,249
*θ*_w_	0.0017	0.0018	21,419	0.0018	0.0019	21,463	0.30	0.0017	0.0015	20,249
*π*_nonsyn_^b^	0.0011	0.0029	20,605	0.0014	0.0033	20,696	0.15	0.0012	0.0025	19,953
*π*_syn_^c^	0.0044	0.0116	20,605	0.0049	0.0343	20,696	0.04	0.0046	0.0182	20,249
*θ*_w nonsyn_^b^	0.0014	0.0029	20,605	0.0017	0.0034	20,696	0.14	0.0012	0.0025	19,953
*θ*_w syn_^c^	0.0047	0.0114	20,605	0.0056	0.0344	20,696	0.04	0.0051	0.0183	19,953
*D*	–0.72	0.89	19,691	–0.63	0.85	19,984	0.03	–0.67	0.66	19,940
*H*	–0.41	1.23	19,574	–0.49	1.28	19,893	0.07	–0.44	0.97	19,911

^a^Values are nucleotide diversity, *π*; polymorphism Watterson’s estimator, *θ*_w_; *D* is Tajima’s *D*; *H* is Fay and Wu’s *H*. Mean and sd (standard deviations) of these statistics were calculated from *n* numbers of homeologs. Correlations between homeolog diversity statistics are shown as Pearson’s correlation coefficient (*r*). All *p*-values for correlations are <0.0001

^b^nonsyn = non-synonymous substitutions, ^c^syn = synonymous substitutions

Higher proportions of non-synonymous mutations were found to be at low frequency compared with synonymous mutations and no significant differences in the relative proportions were found between subgenomes (Fig. 1b). This suggests purifying selection on a large proportion of amino-acid-changing substitutions in both subgenomes. Frequency-based test statistics clearly show significant departures from neutrality for both subgenomes (Fig. 1c). The mean values of Tajima’s *D* were negative for both subgenomes (Table 2, Fig. 1c) owing to high proportions of rare variants.

The distributions and means of Tajima’s *D* in *A. kamchatica* (Table 2) are similar to early genome-wide data from *A. thaliana* (mean Tajima’s *D*_*A. thaliana*_ = −0.8)^41^, though more recent estimates using over 300 genomes show a higher mean but not higher median in *A. thaliana* (mean *D*_*A. thaliana*_ = 0.006, median *D*_*A. thaliana*_ = −0.33)^31^, which likely reflects more intermediate-frequency polymorphisms in the large species-wide sample. The same study reported an excess of rare variants in the diploid relatives of *A. kamchatica* (mean *D*_*A. lyrata*_ = −0.99 and *D*_*A. halleri*_ = −0.23)^31^.

We found the means of the distributions for most summary statistics to be very similar between the two subgenomes, but when pairs of all homeologs were compared, correlations were generally low for diversity and neutrality estimators (Table 2). The correlations of *π*_syn_ and *θ*_w syn_ were both nearly zero (Table 2). Similarly, Tajima’s *D* shows overlapping distributions and similar means for both subgenomes, but the correlation for Tajima’s *D* between pairs of homeologs was very low (*r* = 0.03). The Fay and Wu’s *H* statistic, which detects departures from neutrality due to intermediate and high-frequency variants, also shows a very low correlation between homeologs (Table 2). Higher correlations were observed for non-synonymous or total sites, but this can be explained by the constraints on non-synonymous changes.

To test whether the low correlations in the homeologs were inherited from the parental diploid species, we also compared the orthologs of *A. halleri* and *A. lyrata*. Tajima’s *D* also showed a low correlation (*r* = 0.08), but much higher correlations in genetic diversity statistics (*π*_nonsyn_ and *π*_syn_) were observed (*r* = 0.43–0.54; Supplementary Table 6) compared with those between the homeologs. Therefore, the low correlations in the homeologs are consistent with different evolutionary trajectories of individual duplicated pairs.

### Effective population sizes and species divergence estimates

We calculated *N*_e_ using our empirical estimates of nucleotide diversity (*π*) for *A. kamchatica* and both diploid species and two different mutation rates^42,43^ and found the values for the polyploid were several times lower than those for the diploid species (Supplementary Table 7). We ran coalescent simulations of two demographic models (simple divergence model M1 and population expansion model M2) with intergenic SNPs (Supplementary Table 8) using fastsimcoal2^44^ to estimate the relative *N*_e_ and population split times (*Tdiv*) of each subgenome from their corresponding diploid species (Supplementary Table 9). Likelihood ratio tests showed that the best-fit model was a simple divergence model, M1. For the two models, the *N*_e_ of the *halleri*-origin subgenome of *A. kamchatica* was estimated to be 74–103k, and very similar estimates were obtained using the *lyrata*-origin subgenome (74–99k); the estimates of *N*_e_ of diploid parental species were several times higher (307–407k for *A. halleri* and 314–371k for *A. lyrata*). The estimates of *N*_e_ using fastsimcoal for each species was consistent with those using nucleotide diversity. The *Tdiv* estimates of each subgenome from the diploid species were similar for model M1 (H-origin: 87–105k years, L-origin: 121–145k years, where *k* = 1000) and model M2 (H-origin: 64–79k years, L-origin: 77–99k years). These estimates also agree with a previous report using just two nuclear loci^28^. We interpret the estimates of *N*_*e*_ and *Tdiv* with caution, as the mutation rates for these species have not been estimated directly, simple models were assumed, and the diploid samples were limited.

### Mean rate of LD decay in both subgenomes

Long scaffold assemblies allowed us to estimate genome-wide LD for each subgenome to evaluate the feasibility of association mapping in *A. kamchatica*. The LD in both subgenomes decreased drastically within 5–20 kb, and then *r*^2^ decreased gradually over tens of kilobases (Fig. 1d). The mean LD decay for the *lyrata* subgenome decayed slightly faster but remained asymptotic at *r*^2^ = 0.47 over the scale of 100 kb (Supplementary Fig. 2A), while the mean LD decay for the *halleri* subgenome remained asymptotic at *r*^2^ = 0.34 at 100 kb. The 50 and 90% confidence intervals around the mean LD decay indicated enormous variance in LD for both subgenomes, but greater variance in the *lyrata* subgenome was observed (Supplementary Fig. 2B).

Population structure assignments and phylogenetic clustering (Supplementary Note 3) may provide some explanation for subgenome differences in the level of LD. Over long distances, the mean *r*^2^ decay curve approaches its minimum level, which is often determined by relatedness within subpopulations, or population structure (for example, *r*^2^ between 0.1 and 0.6 beyond 20 kb for different populations of *A. thaliana*)^45^. The 25 *A. kamchatica* accessions cluster geographically with one main clade/group comprising the northern accessions (Russia, Sakhalin, and Alaska) and the other main group containing Japanese accessions (Supplementary Figs. 3 and 4), which is part of the southern end of the species distribution. The branch lengths within these groups for the *lyrata* subgenome are shorter than for the *halleri* subgenome, particularly within the Japanese clade. This suggests greater relatedness among the *lyrata*-origin haplotypes within the two main geographic regions, but higher divergence between the northern and southern accessions. The clusterings are also consistent with previous haplotype analysis using low-density nuclear and chloroplast markers^29^.

### Diversity of the *HMA4* locus and the genomic background

**Fig. 2:**
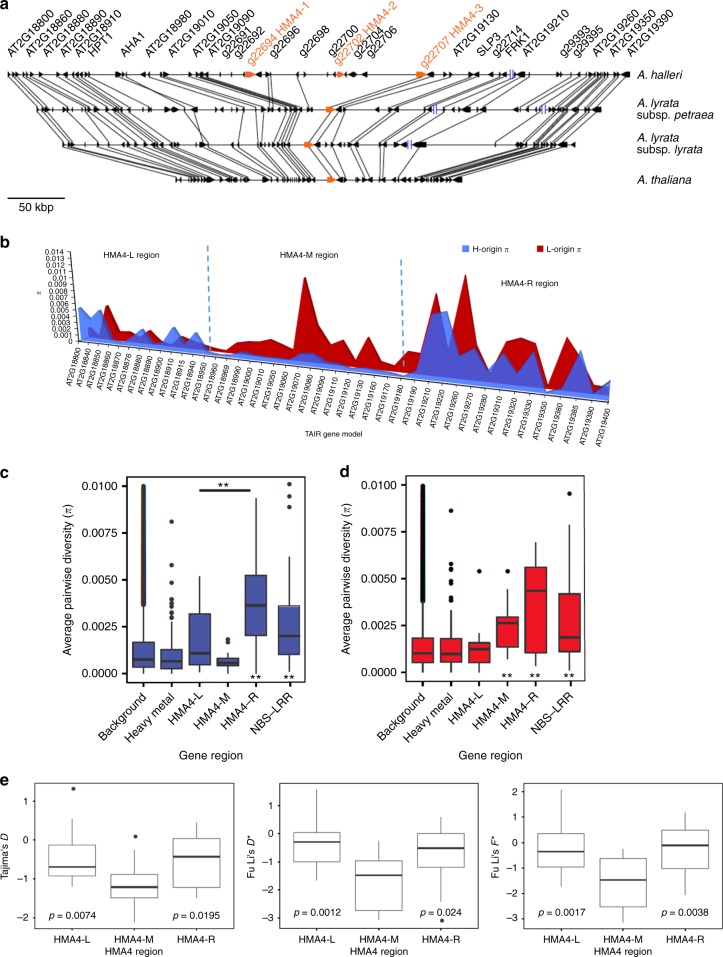
Genetic diversity of the syntenic *HMA4* region. **a** Synteny of the *HMA4* region from *A. halleri* v2.2^38^, *A. lyrata* subsp. *petraea* v2.2, *A. lyrata* subsp. *lyrata* (JGI)^70^, and *A. thaliana* (TAIR). **b** Nucleotide diversity (*π*) of genes surrounding the *HMA4* region in both homeologs of *A. kamchatica*. **c** For the *halleri* subgenome, genetic diversity of NBS-LRRs is significantly greater (two asterisks below, **p < 0.001, Wilcoxon signed-rank test) than the background genetic diversity, whereas heavy metal (HM) genes show no significant difference. Diversity for both HMA4-L (*π* = 0.0018) and HMA4-R (*π* = 0.004) are significantly higher than the HMA4-M (which contains the *HMA4* coding sequences) region (two asterisks above HMA4-M, **p < 0.001, Wilcoxon signed-rank test). **d** For the *lyrata*-subgenome, diversity of NBS-LRRs, HMA4-M, and HMA4-R are all significantly higher than the background. The diversity of the *lyrata* HMA4-M (*π* = 0.0032) region is also significantly greater than the *halleri* HMA4-M region (*π* = 0.0007, paired *t*-test *p*-value = 0.003; Wilcoxon signed-rank *p*-value = 0.0001). **e** The neutrality statistics (Tajima’s *D*, Fu and Li’s D*, and Fu and Li’s F*) all show the *halleri*-origin HMA4-M region to be significantly lower than the left- and right-flanking regions, supporting genetic hitchhiking surrounding the *HMA4* coding sequences. Boxplots show center line: median; box limits: upper and lower quartiles; whiskers: not greater than 1.5 times the interquartile range; points: outliers

We analyzed genetic diversity on the scaffolds containing the *HMA4* locus (Fig. 2a) to compare whether it differs from the genomic background and the surrounding regions flanking the *HMA4* coding sequences. The distribution of *π* in the HMA4-M region (which contains the *HMA4* coding sequences) for H-origin genes showed low diversity (*π*_mean_ = 0.0007), but it is not significantly lower than that of the background genes (Fig. 2b, c). However, the two adjacent regions (HMA4-L and HMA4-R) compared with the HMA4-M (containing the *HMA4* coding sequences) region have significantly greater diversity (Fig. 2b, c). Furthermore, we found significantly lower Tajima’s *D*, Fu & Li’s D* and Fu & Li’s F* statistics in the HMA4-M region compared with both adjacent regions (Fig. 2e), suggesting greater selection on the HMA4-M region. The significantly lower diversity and neutrality statistics in HMA4-M compared with the adjacent regions likely defines the window of the sweep region previously reported for *A. halleri*^39^.

Unlike the *halleri* HMA4-M region, the diversity of the *lyrata* HMA4-M region is significantly greater than the genomic background (*p*-value = 0.0028, Wilcoxon signed-rank test), but not different from the two adjacent regions (Fig. 2d). Moreover, the *lyrata*-HMA4-M region shows no significant differences from the adjacent HMA4-L or HMA4-R regions for Tajima’s *D* (HMA4-L vs. HMA4-M: *p*-value = 0.38 and HMA4-M vs. HMA4-R *p*-value = 0.73, Wilcoxon signed-rank test), Fu & Li’s D* (HMA4-L vs. HMA4-M: *p*-value = 0.86 and HMA4-M vs. HMA4-R *p*-value = 0.36, Wilcoxon signed-rank test), and Fu & Li’s F* (HMA4-L vs. HMA4-M: *p*-value = 0.40 and HMA4-M vs. HMA4-R *p*-value = 0.38, Wilcoxon signed-rank test). The elevated diversity of the *lyrata*-origin *HMA4* locus compared with the genomic background is consistent with relaxed selective constraint on the *lyrata*-origin *HMA4* locus.

We also estimated diversity of all annotated heavy metal transporters, metal ion transporters, and metal homeostasis genes for comparison with the genome-wide average (HM genes, *N* = 118 genes). We expected these genes to have low overall diversity in both genomes due to selective constraint, as many of these ion transporters are expected to have roles in basic metal homeostasis (Supplementary Methods). As a contrast, we compared NBS-LRR genes (*N* = 39 genes), which have putative roles in plant defense and high diversity in plants, and which are expected to have equally high diversity in both subgenomes. The HMA4-L and HMA4-R regions in both subgenomes have more similar levels of diversity to NBS-LRR levels than to those of the genomic background or HM genes (Fig. 2c, d).

### Selection on homeologous proteins

**Fig. 3:**
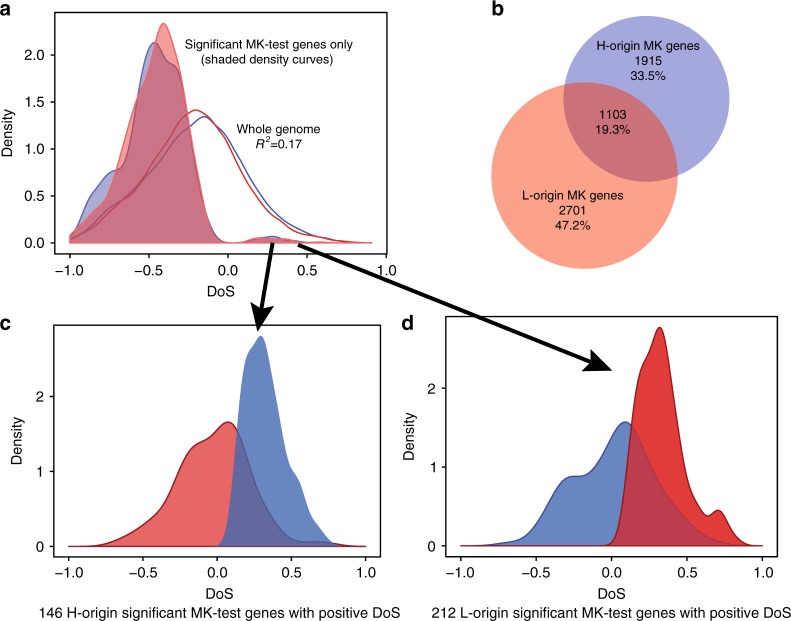
The direction of selection for both subgenomes. **a** Density curves of the direction of selection (DoS)^14^ for about 21,000 coding sequences (blue line and density curves are DoS for H-origin genes, red line and density curves are DoS for L-origin genes). Neutral genes are indicated by 0, while negative values indicate purifying selection and positive values indicate positive selection. The means of these distributions are −0.20 and −0.22 for the H- and L-origin homeologs, respectively, and show that ~70% of both homeologs have a negative selection index (negative DoS). Shaded density curves are genes that were significant for MK tests (*p* < 0.05 using Fisher’s marginal *p*-values). **b** Only 19% of genes show significance for MK tests for both homeologs. **c** Using only significant MK-test genes with positive DoS for *halleri*-origin and **d** positive DoS for *lyrata*-origin genes show that the other homeolog has significantly more negative DoS (*p*-value < 2.2*e*−16 using pairwise *t*-test and Wilcoxon signed-rank test) when one shows positive selection using comparisons of DoS distributions in both **c** and **d**

Next, we used divergence–diversity tests to estimate the strength of purifying and positive selection on amino-acid changing substitutions. We calculated the divergence of each homeolog from the outgroup *A. thaliana* to estimate the relative proportions of diverged non-synonymous (*D*_n_) and synonymous (*D*_s_) sites to polymorphic non-synonymous (*P*_n_) and synonymous (*P*_s_) sites. For each gene, the counts of *D*_n_, *D*_s_, *P*_n_, and *P*_s_ for the coding regions of both subgenomes were used to estimate the DoS^14^, a neutrality index that varies from −1.0 to 1.0, where zero indicates neutrality and negative and positive values indicate purifying and positive selection, respectively. Both subgenomes had DoS with means of −0.2 and similar distributions (Fig. 3a), suggesting that 68–71% of proteins derived from both subgenomes are under purifying selection (when DoS is <−0.01). Like the previous summary statistics, the correlation in DoS between *halleri* and *lyrata* homologs is positive but fairly low (*R*^2^ = 0.17).

MK tests were conducted to detect purifying selection or adaptive evolution on amino-acid changing mutations. Among the significant MK-test genes, a total of 3018 H-origin and 3804 L-origin homeologs showed DoS < 0 (*D*_n_/*D*_s_ < *P*_n_/*P*_s_). This is consistent with purifying selection rather than positive selection for these genes. While the homeologs with significant MK-test results comprise a substantial portion in our dataset, only 19% of them include both homeologs (i.e., there is significance for one homeolog but not the other for 81% of significant homeologous pairs, Fig. 3b). For example, the H-origin homeolog of the resistance gene *RPM1* (orthologous to *A. thaliana* gene: AT3G07040) was significant for the MK test (DoS < 0) but the L*-*origin copy was not.

Among genes showing positive selection (or adaptive evolution) using MK tests, 146 *halleri*-origin and 212 *lyrata*-origin genes were significant when DoS > 0.01 (Fig. 3c, d). For these genes, when the *halleri*-derived homeolog shows a positive DoS, the *lyrata*-derived homeolog shows a more neutral or negative distribution in DoS and vice versa. Among these is the H-origin *HMA4* gene. These results, in addition to the low correlation in DoS for pairs of homeologs and small overlap among all significant MK-test genes (Fig. 3b), indicate that a substantial proportion of homeologs have been shaped by different strengths of selection. These results are also in agreement with low correlations in Tajima’s *D* and Fay and Wu’s *H* for pairs of homeologs (Table 2), providing additional support that homeologous genes exhibit significant differences due to stronger positive or purifying selection on only one of the two copies.

### The distribution of fitness effects

**Fig. 4:**
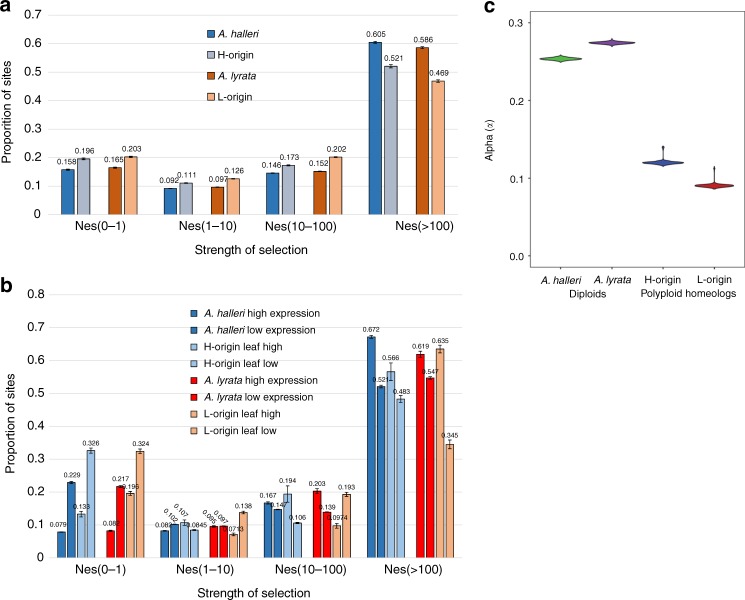
The strength of purifying selection and adaptive evolution. **a** The distribution of fitness effects (DFE) of deleterious mutations for coding sequences of the two *A. kamchatica* subgenomes and corresponding diploid orthologs of *A. halleri* and *A. lyrata*. The strength of selection is indicated by *N*_e_*s* where *N*_e_ is the effective population size and *s* is the selection coefficient. Error bars show sd. **b** DFE categorized by expression in both subgenomes and diploid species. Expression categories were taken from the upper 10% (high) and lower 10% (low) of expression distribution in all *A. kamchatica* homeologs. The DFE was estimated from 10^6^ MCMC replicates. Error bars are sd from 1000 resampled MCMC replicates. **c** The proportion of adaptive non-synonymous substitutions (*α*) for both subgenomes (H-origin *α* = 0.12, CI: 0.117–0.141, L-origin *α* = 0.09, CI: 0.087–0.094) and for the two corresponding diploid species (*A. halleri* *α* = 0.25, CI: 0.251–0.257, *A. lyrata* *α* = 0.27, CI: 0.272–0.277) are significantly greater than zero

The tests above indicated that large numbers of homeologs show patterns consistent with purifying selection on amino-acid changing mutations (Fig. 3). We quantified the genome-wide proportions of deleterious and effectively neutral mutations using the DFE method^13^ in the two *A. kamchatica* subgenomes and both diploid relatives. In this method, the DFE is estimated from the SFS of non-synonymous and synonymous polymorphisms while accounting for the effects of demographic changes. Effectively neutral mutations are represented by 0 < *N*_e_*s* < 1, mildly deleterious by 1 < *N*_e_*s* < 10, deleterious by 10 < *N*_e_*s* < 100, and strongly deleterious by *N*_e_*s* > 100 (where *N*_e_ is the effective population size and *s* is the selection coefficient). The DFE estimates of the two *A. kamchatica* subgenomes show similar distributions, with about 70% of mutations in the deleterious to strongly deleterious categories (*N*_e_*s* > 10) and 20% effectively neutral (0 < *N*_e_*s* < 1) (Fig. 4a). The DFE of *A. halleri* and *A. lyrata* showed lower proportions of neutral mutations (16–17% of mutations 0 < *N*_e_*s* < 1 in diploids, and 20% mutations 0 < *N*_e_*s* < 1 in both subgenomes) and greater proportions of deleterious mutations (*N*_e_*s* > 100) than either of the corresponding allopolyploid subgenomes. These differences are significant but not extreme.

We also expected genes that are highly expressed to show the strongest purifying selection, and lowly expressed genes to show relaxed constraint^46^. Consistent with this expectation, we found that evolutionary rates (dN/dS) in both homeologs were negatively correlated with gene expression (Supplementary Fig. 5A and 5B). We estimated the DFE again, but using expression levels in *A. kamchatica* to categorize homeologs as having high (genes in upper 10% of RPKM) or low expression (lower 10% of RPKM). The DFE patterns indicated that lowly expressed genes had the highest proportion of neutral mutations (relaxed constraint) (33% in H-origin subgenome, 0 < *N*_e_*s* < 1) and the lowest proportion of deleterious mutations (48% in H-origin subgenome, *N*_e_*s* > 100), while highly expressed genes showed the opposite pattern (13% and 57%, respectively) (Fig. 4b). The same pattern was found using expression data from either leaf or root tissue (Supplementary Fig. 5C). Like the *A. kamchatica* homeologs, for the two diploid species, genes with high expression showed lower proportions of neutral substitutions (relaxed constraint) and higher proportions of strongly deleterious substitutions compared with lowly expressed genes. Therefore, within each species, gene expression predicts relaxed constraint and the DFE method can detect relaxed constraint and strong purifying selection as predicted when gene expression levels are taken into account^47^.

To further examine differences in relaxed selection, we compared the diploid species and corresponding subgenomes in high and low expressed genes (Fig. 4b) and again found significant differences between the diploids and polyploid. Genes that had low expression in the diploids had significantly lower proportions of neutral mutations than the low expressed homeologs (0 < *N*_e_*s* < 1: 23% in *A. halleri* and 33% in H-origin, and similar for the *A. lyrata*–L-origin comparison; Fig. 4b). Likewise, the highly expressed genes in the diploids showed a significantly lower proportion of neutral mutations than the highly expressed homeologs in *A. kamchatica* (0 < *N*_e_*s* < 1: 8% in *A. halleri* and 13% in H-origin, and similar for the *A. lyrata*–L-origin comparison; Fig. 4b). The difference in these subsets appears more pronounced compared with that of the genome-wide data where a large number of genes were averaged. These results support the trend also found in the genome-wide data, that the outcrossing ancestral species with greater *N*_e_ have fewer neutral mutations and higher proportions of deleterious mutations than their derived, self-fertilized subgenomes.

### The proportion of adaptive substitutions

The proportion of adaptive substitutions (*α*) was estimated as the excess of between-species divergence relative to polymorphism as expected from the estimated DFE^13^ to account for slightly deleterious mutations. In contrast to the majority of the previously studied plant species including *A. thaliana*, we found significantly positive values of *α* for the two diploid species and both allopolyploid subgenomes. The diploid species *A. halleri* and *A. lyrata* showed the highest *α* values (0.25 and 0.27, respectively) (Fig. 4c). We subsampled 18 *A. kamchatica* accessions to be statistically comparable to the available *A. halleri* and *A. lyrata* samples (Supplementary Table 5). The *α* estimates for the H- and L-origin subgenomes of *A. kamchatica* were lower than those of the corresponding diploid species but significantly greater than zero (0.12 and 0.09, respectively) (Fig. 4c). The difference in *α* between subgenomes was significant but subtle (3% using the samples above, and 6% difference when all 25 *A. kamchatica* accessions were used; Supplementary Fig. 6).

### High-impact mutations are at low frequency in both subgenomes

We identified genes having high-impact mutations that are likely to be deleterious due to their putative effects on amino-acid sequences and gene expression into the following mutation categories: frameshifts, loss-of-start codon, premature stop codons (stop-gained), and loss-of-stop codons (stop loss). For any gene, we counted every one of the mutation types regardless of the number. While it is not possible to determine the order of disruptive mutations, multiple frameshifts or premature stop codons in a gene would be expected to result in a loss of function.

**Fig. 5:**
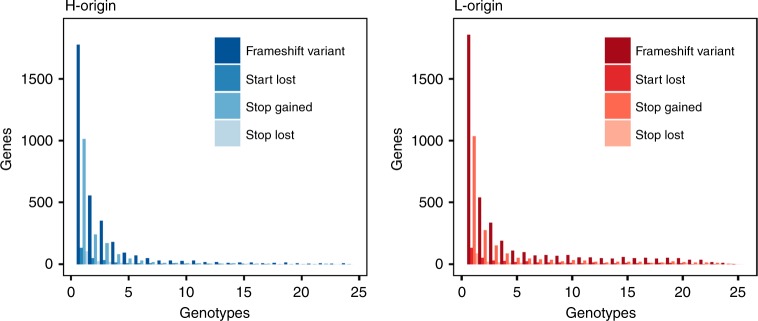
Frequency distributions of high-impact mutations. The majority of large-effect mutations are at low frequency for both subgenomes. The *x* axis is the number of genotypes in our resequencing collection (25) and the *y* axis is the frequency of genes in each of the four categories of large-effect mutations

Frameshifts and stop-gained categories comprised the majority of mutation types for both subgenomes (Supplementary Table 10, Supplementary Note 4). Frequencies of each mutation type indicated that most mutation types in any gene are found in only a single genotype in either subgenome (Fig. 5). There were slightly greater proportions at low frequency in the *halleri*-homeologs. Enriched gene ontology (GO) categories included nucleotide binding and catalytic activity (Supplementary Table 11). Out of the total 4219 *halleri*-origin and 4952 *lyrata*-origin disrupted genes, only 511 genes (2.5%) showed large-effect mutations in both homeologs in the same accession, suggesting that large-effect mutations in both homeologs were highly deleterious. The distribution of genes with high-impact mutations in both homeologs shows that most accessions have <50 genes (orthologous to *A. thaliana*) that are disrupted with putatively similar functions (Supplementary Fig. 7). Enriched GO categories with high-impact mutations in both homeologs in a single accession included transducer/receptor activity and programmed cell death (Supplementary Table 11).

## Discussion

A recurrent pattern we observed while analyzing patterns of diversity and signatures of selection was that the genome-wide distributions were similar between subgenomes, but the correlations between the pairs of homeologs were low. We found this pattern in the polymorphism levels such as *π*_syn_ and *θ*_w syn_, in frequency-based tests of neutrality (Tajima’s *D*, Fay and Wu’s *H*), and in divergence–diversity-based tests (DoS). Because both subgenomes have experienced identical demography since hybridization, the much lower correlations in *π*_nonsyn_ and *π*_syn_ in the polyploid compared with the diploid species suggests that a large number of homeologs have been shaped by different levels of selection and relaxed constraint due to genome duplication. This also supports the hypothesis that homeologs may evolve as independent loci, which may not be surprising because *A. kamchatica* shows disomic inheritance prohibiting recombination between homeologs^28^. These results also suggest that the difference between homeologs could contribute to the broad environmental response of polyploids, which may be realized by combining different adaptations of two parental species^10^ such as in the *HMA4* gene.

Long scaffolds containing the *HMA4* copies and surrounding genes allowed us to compare homeologs across large genomic distances. The genetic diversity surrounding the *halleri*-derived *HMA4* gene that spans ca. 300 kb (HMA4-M) is significantly lower than the syntenic *lyrata*-derived region (ca. 100 kb), suggesting different evolutionary pressures or trajectories of functional duplicates. The higher diversity of the *lyrata* HMA4-M region is consistent with a pattern of relaxed constraint, while a selective sweep and genetic hitchhiking characterize the *halleri-*derived HMA4-M region. Despite multiple hybrid origins of *A. kamchatica*, the tandem triplication (three *halleri*-derived *HMA4* copies) is fixed in the allopolyploid^10^, suggesting it was present in all founding *A. halleri* parents. Because we can infer that the triplication was ancestral and the reduced diversity at this locus and hitchhiking surrounding the *HMA4* genes were most likely the result of strong selection in the *A. halleri* parent^39^, diversity was probably greatly reduced prior to the polyploidization events.

We used the DFE method to quantify the proportions of deleterious mutations. Theoretical studies suggested that higher proportions of neutral mutations (i.e., greater relaxed constraint) can result from whole-genome duplication due to the reduction of *N*_e_ or due to the masking of deleterious mutations by functionally redundant gene copies^15,16^. This would be evident through the existence of greater proportions of effectively neutral mutations (0 < *N*_e_*s* < 1) in the polyploid subgenomes compared with the diploid parents^8^. Similarly, greater proportions of deleterious mutations (*N*_e_*s* > 10) in the diploid species would be expected compared with their derived polyploid subgenomes. We did detect significant differences between diploid parental species and the corresponding subgenomes of *A. kamchatica* in the proportions of mutations in the neutral (<5% differences) and deleterious to strongly deleterious (5–7% differences) categories, although the differences did not appear drastic (Fig. 4a).

Using a similar approach, the change in relaxed constraint and purifying selection was estimated by comparing the allopolyploid species *C. bursa-pastoris* with its diploid parents, *C. grandiflora* (outcrossing species with *N*_e_ = ca. 800,000) and *C. orientalis* (selfing species with *N*_e_ = ca 5000)^8^. Accordingly, neutral mutations doubled from ~17% in the *C. grandiflora* parent to ~35% in the *C. grandiflora*-derived subgenome. Because the *N*_e_ of the derived subgenome of *C. grandiflora* was estimated to be 20 times smaller (*N*_e_ = ca. 40,000) than the diploid parent^8^, this demonstrated that the subgenome derived from an outcrossing parent with a large *N*_e_ shows a high proportion of neutral mutations due to relaxed constraint. The opposite pattern was observed in the subgenome derived from the highly inbreeding parent, which showed a decrease from ~40% to ~35% neutral mutations due to an increase in *N*_e_ in the polyploid compared with the selfing parent. The difference in relaxed constraint between *A. halleri* and *A. lyrata* and the two subgenomes of *A. kamchatica* appear less extreme than in the *C. bursa-pastoris* and *C. grandiflora* comparison, likely due to less extreme differences in our estimates of *N*_e_ between *A. halleri*, *A. lyrata*, and *A. kamchatica*, which are about 4–5 times less *N*_e_ for the subgenomes than the diploids (Supplementary Tables 7 and 9). These results, along with our genetic diversity analysis, suggest that the bottleneck associated with polyploidy in *A. kamchatica* may not have been so severe.

When genes were categorized by expression level, we found even greater differences in neutral mutations between the diploid species and the polyploid subgenomes compared with the genome-wide data. For genes with low expression, the polyploid subgenomes had about 10% more neutral mutations compared with the lowly expressed genes in the diploid species. A similar level of difference in neutral mutations between the polyploid and diploids was found for highly expressed genes. Overall, our results are consistent with expectations of relaxed constraint on genes with low expression and stronger purifying selection on highly expressed genes^46^ in all three species. However, the smaller *N*_e_ of the polyploid, inbreeding, and increased potential for masking non-synonymous mutations in duplicated genomes contributes to greater relaxed constraint compared with the diploid progenitors^48^.

Previous multispecies comparisons showed that only a few plant species have *α* values that are greater than zero^17^, but those estimates were mostly done using limited genetic data (<1000 loci)^17,19^ rather than genome-wide data. We estimated that 25–27% of non-synonymous substitutions are adaptive in the two diploid species *A. halleri* and *A. lyrata*. These are the highest estimates of *α* for any *Arabidopsis* species^17,40^ and higher than for most plant species. The highest *α* among any plant species was estimated in the highly outcrossing *C. grandiflora* (*α* = 0.4–0.7)^19,49^, with levels similar to those of *Drosophila* and bacteria—all taxa with large effective population sizes^18^. Our results for the diploid species are consistent with those of previous studies that have shown a positive correlation between *α* and *N*_e_^17,20^; this suggests that greater adaptive evolution often occurs in species with large effective population sizes. This is true for both highly outcrossing diploid species reported here.

Importantly, *α* for both subgenomes of *A. kamchatica* is also significantly greater than zero and indicates that 6–12% of non-synonymous substitutions are adaptive. Many diploid plant species have a similar or larger effective population size than *A. kamchatica*, but do not show positive *α*^17^. For example, *N*_e_ estimated for *A. thaliana* was between 65,000 and 267,000^17,20^ while *α* = −0.08^19^, indicating that effective population size alone cannot explain the significantly positive *α* of *A. kamchatica*. These data suggest that *A. kamchatica* has a positive *α* because of polyploidy. We propose two mutually non-exclusive explanations. First, *A. kamchatica* may have inherited fixed non-synonymous or adaptive substitutions from the two parental species. The *α* values of *A. kamchatica* are roughly half of the parental species, in which the reduction may be attributable to the reduction of *N*_e_. Second, non-synonymous mutations are increased at the early stages of polyploid species due to masking of duplicate copies, in contrast to the slow rate of non-synonymous mutations in more ancient duplicated genes^21,22^. A classic idea of the high evolvability of duplicated genomes states that one of the duplicated copies may be able to obtain a new function or adaptive mutation because the other copy retains the original function^23,24^.

The loss of homeologs in ancient polyploids, or non-functionalization, has been extensively studied^24^, but relatively little is known about the population genetics of young polyploid species. We identified high-impact mutations that are likely to disrupt the gene function. We found that about 20% of the homeologs in both subgenomes had disruptive mutations in our collection of 25 individuals (Supplementary Table 10), although their frequencies are low (Fig. 5), and only rarely are both homeologs disrupted. Interestingly, we found that high-impact mutations were rarely fixed. Genes with high-impact mutations may not be annotated as genes in a direct assembly of a polyploid genome, so the use of diploid reference genomes in our methods should be sensitive in detecting high-impact mutations even if fixed in polyploids. The paucity of fixed mutations is in contrast with the results from another allopolyploid species, *C. bursa-pastoris*, in which a large proportion of high-impact mutations (such as stop codon gained) were fixed^8^. In *A. kamchatica*, similar proportions of high-impact mutations were at low frequency compared with non-synonymous substitutions, which were also at low frequency (Fig. 1b), suggesting that genome-wide purifying selection keeps their frequency low. This is consistent with the prevalence of purifying selection shown by DoS and by DFE methods.

Drastic improvements in the genome assembly of polyploid crop species with large genomes^50–52^ will facilitate genome-wide polymorphism analyses and scans for selection. Whole-genome duplication may allow duplicated genes to obtain new functions or adaptive mutations because the other copy retains the original function^23,24^. Although theoretical analysis typically assumes that deleterious mutations may be masked by genome duplication, empirical studies showed that the dosage balance in gene networks may be a selective constraint^53^ and could work as a mechanism for purifying selection in allopolyploid species. By quantifying selection using polyploid species with different population sizes, times since polyploidization, and mating systems, general patterns of selection in polyploid genomes will emerge.

We found that the level of nucleotide diversity of *A. kamchatica* is moderate and similar to that of the diploid self-compatible *A. thaliana*, but six times lower than the diploid outcrossing species *A. halleri* and *A. lyrata*. The extent of LD decay in *A. kamchatica* is comparable to the self-fertilizing species *A. thaliana*^45^ and *M. truncatula*^54^ and appears adequate for characterizing the genetic architecture of complex traits within relatively narrow genomic windows using genome-wide association studies (GWAS). The self-fertilizing mating system, levels of genetic diversity, LD, and a recently established transgenic technique^55^ suggest that *A. kamchatica* would be a suitable model for functional genomics of adaptive mutations in a polyploid species. A further step will be to incorporate polymorphism, gene expression, and species distribution data (i.e., landscape genomics) of diploid parents and allopolyploid hybrids to identify the contributions of parental adaptations for broadening climatic regimes and abiotic habitats in polyploids.

## Methods

### Allopolyploid plant samples and resequencing

*Arabidopsis kamchatica* (Fisch. ex DC.) K. Shimizu & Kudoh^27^ is an allotetraploid species distributed in East Asia and North America. We consider Russian individuals described as *Cardaminopsis kamtschatika* or *Cardaminopsis lyrata* as synonyms (note that *A. lyrata* is a distinct diploid species)^56^. Genomic DNA from 25 accessions of *A. kamchatica* was extracted from leaf tissue using the DNeasy Plant Kit (Qiagen). These accessions were collected from Taiwan, lowland and highland regions of Japan, Eastern Russia, Sakhalin Island, and Alaska, USA (listed in Supplementary Table 3). DNA concentration and quality were measured using Qubit. Genomic DNA libraries were constructed at the Functional Genomics Center Zurich (FGCZ) using NEB Next Ultra. Total DNA was sequenced on Illumina HiSeq 2000 using paired-end sequences with an average insert size of 200–500 bp. Read lengths were 100 bp.

### Read sorting using diploid reference genomes

De novo genome assemblies of *A. halleri* and *A. lyrata* were used for assigning sequencing reads from polyploid individuals to their parental origins (see Supplementary Methods for genome assembly information). Illumina reads from *A. kamchatica* were mapped using BWA-MEM version 0.7.10 on the two diploid genomes independently. We classified the reads to each parental origin as H-origin (*halleri*-origin) and L-origin (*lyrata*-origin) using HomeoRoq (http://seselab.org/homeoroq)[32]. In this method, reads from each accession were first mapped to each parental genome, and then classified as H-origin, L-origin, common, or unclassified (see Fig. 1 in Akama et al.^32^ for a schematic diagram). Here, the common reads are the reads that aligned equally well to both parental genomes. After mapping to the *A. halleri* genome, we detected *A. kamchatica halleri*-origin (H-origin) reads and identified single-nucleotide polymorphisms (SNPs) and short insertions and deletions using GATK v3.3^57^. Then, the nucleotides were replaced on the detected variant position in the reference genome with the alternative nucleotides if the position (1) had coverage of least 20% of the average coverage by reads in each library, (2) covered by at most twice of the average coverage, and (3) had 30 or higher mutation detection quality (QUAL) reported by GATK. This cycle of mapping, read classification, and reference modification was repeated 10 times. For the reference modification, we used only origin reads the first five times and both origin and common reads the last five times. The *A. kamchatica lyrata*-origin (L-origin) genome was iteratively updated in a similar manner. The modified genomes were only used for read sorting. Coverage was calculated for both subgenomes of our resequenced lines by using the sum of the diploid parents as the genome size (250 + 225 = 475) and common plus sorted origin reads (Supplementary Table 3).

### Variant calling

For final variant calling, we combined the common reads of *A. kamchatica* with sorted H-origin or L-origin reads and aligned them back to the original parental genomes using BWA-MEM v0.7.10. We called variants using GATK v3.3-0 following established best practices^58,59^. We processed each alignment BAM file separately to fix mate pairs, mark duplicates, and realign reads around indels. Then we identified variants by running HaplotypeCaller jointly on all genotypes but separately for each parental subgenome. To remove low-quality variants, we mostly used the thresholds recommended for variant datasets where quality score cannot be recalibrated^59^. We applied quality by depth (QD < 2), mapping quality (MQ < 30), MQ rank sum (MQRankSum < −15), and genotype quality (GQ < 20) filters. Because some of our accessions had relatively low coverage, we considered that the recommended strand and read position filters might be too strict and we did not apply them. We filtered variants that GATK reported as heterozygous and replaced them as Ns. We expected this to have very minor effects on our analyses^54,60^ because *A. kamchatica* is highly self-fertilizing^61^ and self-compatible and most residual heterozygosity is expected to be removed from our germplasm. We used diploid data from nine accessions of Eurasian *A. halleri* and nine accessions of European *A. lyrata* from Novikova et al.^31^ (see Supplementary Table 4 for accession numbers) mapped to our diploid reference genomes and called SNPs using the same criteria. The diploid VCF files were then phased using Beagle^62^ to produce 18 alleles for each species.

Because regions with excessively high coverage are likely to be repetitive or incorrectly assembled, variants called in those regions are probably spurious. To determine the coverage thresholds, we summed up the coverage reported by BamTools^63^ for each position in the final alignment files across all genotypes. We only considered reads with MQ of at least 20. Then, we calculated the mean and sd for the distribution of the obtained sums in each parental genome. We assumed a Poisson distribution and added 5 sd to the mean to determine the thresholds. These thresholds (2891 and 2509 for *A. halleri* and *A. lyrata*, respectively) were applied to the DP property (total depth of coverage across all genotypes) in the INFO field of the corresponding VCF file. In addition, we applied a coverage filter at the genotype level to exclude calls with coverage below 2 or above 250.

To check for additional spurious variants, we randomly sampled 20 million reads (10 million per parent) from *A. halleri* and *A. lyrata* short-insert (200 bp) reads and ran them through the same variant calling pipeline as the *A. kamchatica* genotypes. The only difference between the runs was that this simulated sample was processed alone whereas variants for *A. kamchatica* genotypes were called jointly. Any variants called with the simulated sample would be due to incorrect read sorting between the parents or repetitive sequences present in the parental genomes. Such spurious variants would also be likely to appear among *A. kamchatica* variants even if the corresponding regions were completely conserved between *A. kamchatica* and its parents. Among the uncovered variants, 59,856 and 58,645 were also present in *A. kamchatica* on the *A. halleri* and *A. lyrata* sides, respectively. All of these variants were marked as filter failing. When applying polymorphisms to the reference sequences, we used Ns in positions where clear calls could not be made due to insufficient coverage, excessive coverage, low-quality polymorphisms, or heterozygosity. Such treatment allowed us to avoid using reference calls in regions where the actual sequence is highly uncertain.

### Coding sequence alignments

We identified homeologous genes based on reciprocal BLAST hits (best-to-best with *E*-values < 10^−15^ and alignment length ≥ 200 bp) among coding sequences from the v2.2 *A. halleri* and *A. lyrata* genome annotations. Using the same approach, we also detected orthologous relationships between the predicted genes in diploid *A. halleri* and *A. lyrata* annotated genome assemblies and *A. thaliana* genes (TAIR 10). In cases of duplicated genes of interest such as *HMA4* (tandemly duplicated three times in *A. halleri*), we used only one copy for diversity analysis due to non-unique alignments of Illumina reads and very high sequence identity (99%) in the *A. halleri* reference genome. Therefore, our genome-wide dataset of coding sequences of homeologs did not contain genes that are duplicated in one genome but not the other.

To make coding sequence alignments, we individually applied SNPs and deletions from each of the 25 *A. kamchatica* genotypes (H-origin or L-origin) to the corresponding reference genomes. For the CDS alignments only, we omitted insertions in order to preserve the genomic coordinates of the coding sequences, which would consequently facilitate the alignment. If a variant was heterozygous, failed the genotype quality filter (GQ < 20), or was not called for a particular genotype (but called for other genotypes), the corresponding bases were replaced with Ns. We assumed that a sequence contains reference bases at positions that are not specified in VCF files and have adequate coverage. Therefore, all bases with coverage <2 (insufficient) or >250 (abnormally high) were replaced with Ns. After that, we extracted coding sequences from the modified genomes and grouped them by gene. Thus, each H-origin or L-origin gene had an alignment file containing 25 aligned coding sequences (one for each genotype). Finally, we aligned *A. thaliana* orthologs as an outgroup using Muscle v3.8^64^ with the profile alignment option, which preserved the alignment of the ingroup sequences and only aligned the outgroup sequence to the core ingroup alignment. The same procedure was used for making gene alignments of the 18 phased alleles for diploid *A. halleri* and *A. lyrata*.

### *HMA4* synteny and sequence alignments

We were interested in comparing homeolog diversity in genes surrounding the *HEAVY METAL ATPASE 4* (*HMA4*) locus. By comparing synteny across multiple *Arabidopsis* species, we can increase the likelihood that homeologs separated by long distances or on multiple scaffolds are indeed syntenic in *A. kamchatica*. We used the published genomes of *A. lyrata* MN47 v1.07 and *A. thaliana* (TAIR, https://www.arabidopsis.org/) which are assemblies of entire chromosomes. We then compared both *A. halleri* W302 and *A. lyrata* v2.2 lyrpet4 regions surrounding the *HMA4* coding sequences. The heavy metal transporter *HMA4* contains three tandemly duplicated ATPase coding sequences in European and Asian *A. halleri*^37,38^. Following synteny analysis of the *HMA4* region, we were then able to examine genetic diversity over longer, contiguous, scaffold regions containing genes flanking *HMA4* coding genes to compare with the genomic background and between subgenomes. We centered the main genomic region containing the *HMA4* genes (three tandem copies in *A. halleri* and *halleri-*origin homeologs and a single *lyrata*-origin copy in *A. kamchatica*), which we call HMA4-M. The region spans 304 kb on H-origin scaffold_0116 and 155 kb in L-origin scaffold_0052. We then used the upstream (left-side) adjacent region (HMA4-L, 125 kb for the H-origin region and 183 kb in the L-origin region), and the downstream adjacent (right-side) region (HMA4-R, 105 kb in H-origin scaffold_0273 region and >50 kb for L-origin region scaffold_0270). We also made alignments for the 118 genes (Fig. 2) with putative roles in metal tolerance, hyperaccumulation, metal ion transport, and metal homeostasis (Supplementary Methods).

### Coding sequence diversity and site-frequency spectra

For gene alignments containing coding sequences, summary and diversity statistics, including divergence from *A. thaliana*, were estimated using libsequence packages^65^ and custom R, Perl, and Ruby shell scripts. The libsequence programs named compute and Hcalc were used to estimate nucleotide diversity (*π*), *θ*_w_, Tajima’s *D*, and Fay and Wu’s *H*. Non-synonymous and synonymous sites were identified using the polydNdS program, which uses the algorithms from Comeron (1995)^66^ with the –P flag to generate SNP tables for each gene. The SFS were created using the SFS.pl program (available from J. Ross-Ibarra’s website http://www.plantsciences.ucdavis.edu/faculty/ross-ibarra/code/files/ea3bd485e4c7dee37c59e8ba77ca800e-11.html) on the set of non-synonymous and synonymous polymorphisms identified using polydNdS. Both folded and unfolded SFS were calculated; the folded spectrum does not differentiate between ancestral polymorphisms and those that are the result of mutations that have entered a population since it split from a common ancestor, while the unfolded spectra are based on derived allele frequencies. We converted the SFS data to SFS count tables using a custom python script (sfs_extraction.py)^47^. We used two published mutation rates, one based on the synonymous substitution rates calibrated by fossil records^42^, and another for total sites in mutation accumulation lines^43^, to estimate the effective population size from our empirical nucleotide diversity estimates using the following equation: *N*_e_ = *π*_syn_ or *π*_total_/4 μ (where *π* was estimated from our data and μ by Koch et al. or by Ossowski et al.^42,43^).

### Demographic analysis

A total of 18 sequences representing single haplotypes from each species were used for demographic analysis (Supplementary Table 5). For *A. halleri* and *A. lyrata*, a total of 18 phased haplotypes were used. For *A. kamchatica*, the H-origin and L-origin sequences derived from 18 genotypes were used as single haplotypes (treated as haploid genomes due to selfing). Because our read sorting and SNP calling method uses two separate reference genomes, two separate two-dimensional joint-site-frequency spectra (2D SFS) composed of minor allele frequencies were generated (Supplementary Table 8) from VCF files using the easySFS program (https://github.com/isaacovercast/easySFS). The *A. halleri*–H-origin subgenome dataset contained 510,649 intergenic sites and the *A. lyrata*–L-origin dataset contained 507,289 intergenic sites. We used fastsimcoal2^44^ to estimate the *N*_e_ of all three species and the divergence time (*Tdiv*) of the H-origin subgenome from *A. halleri*, and separately, the *Tdiv* of the L-origin subgenome from *A. lyrata*. We assumed a mutation rate of 7 × 10^–9^ (Ossowski et al.)^43^ and a generation time of one year. The first model (M1) estimated divergence using a stepwise model of population size change, and the second model (M2) estimated exponential population size changes in the polyploid and diploids. A minimum of 100,000 and maximum of 250,000 coalescent simulations with 10–40 cycles of likelihood maximization were used to estimate parameters and model likelihoods. We compared the two models using likelihood ratio tests to identify the higher model likelihood. We generated 100 simulated joint-site-frequency spectra based on the total numbers of sites in each of the two diploid–subgenome datasets to estimate the 95% confidence intervals of the model parameters.

### Linkage disequilibrium and sliding window diversity

To conduct sliding window analyses along entire scaffolds, we used the PopGenome R^67^ package to calculate diversity of all, intergenic, coding, exonic, and intron regions of *A. kamchatica* using *A. halleri-* or *A. lyrata*-derived VCF and reference gene annotation (.gff) files. We estimated the average nucleotide diversity, Watterson’s *θ*_w_ and *π* (nucleotide diversity, the average number of pairwise nucleotide differences per site). To estimate genome-wide LD, we used the geno-r2 option in VCFtools^68^ across window sizes of a maximum distance of 50 or 100 kb using a minor allele frequency ≥ 0.1, separately for the *halleri-* or *lyrata*-derived VCF files. The resulting *r*^2^ between SNPs were grouped into bins of 50 bp length. We estimated the average, 50%, and 90% confidence intervals of correlation coefficients of each bin.

### Direction of selection and distribution of fitness effects

The program *MKtest* from the libsequence library^65^ was used to count the total number of polymorphic non-synonymous (*P*_n_) and synonymous (*P*_s_) sites in *A. kamchatica* homeologs as well as the number of fixed non-synonymous (*D*_n_) and synonymous (*D*_s_) differences between *A. kamchatica* homeologs and *A. thaliana*. We used the program *MKtest* to perform standard tests on each gene for both homeologs separately; this is a contingency test comparing the numbers of between-species difference and within-species polymorphisms at non-synonymous and synonymous sites where significance is tested using Fisher’s exact tests for each gene.

Polymorphism and divergence data were used to calculate the DoS (DoS = *D*_n_/(*D*_n_ + *D*_s_) − *P*_n_/(*P*_n_ + *P*_s_)) statistic^14^. DoS < 0 is consistent with purifying selection and DoS > 0 is consistent with positive selection. To estimate the DFEs (DFE, i.e., the distribution of the strength of selection acting against new mutations) and the proportion of adaptive substitutions (*α*) in *A. kamchatica*, *A. halleri*, and *A. lyrata*, we used the likelihood method implemented in the software DoFE 3.0^13^. The program was run for 1 × 10^6^ Markov Chain Monte Carlo (MCMC) replicates, and sampled every 1000 replicates after a burn in of 100,000 replicates. To estimate DFE, we used folded allele frequency spectra and the estimated number of non-synonymous (*D*_n_) and synonymous (*D*_s_) differences between *A. kamchatica* homeologs or diploid orthologs and the corresponding outgroup *A. thaliana* orthologs.

### Transcriptome data

We used RNA-seq data collected from leaf and root tissue of the *A. kamchatica* Murodo (Japan) and Potter (Alaska, USA) accessions, and from leaf tissues of *A. halleri* subsp. *gemmifera* (Tada Mine) and *A. lyrata* subsp. *petraea* (lyrpet4) from Paape et al.^10^ to calculate expression for all homeologs in our dataset. We mapped the RNA-seq data to *A. halleri* and *A. lyrata* v2.2 reference genomes and sorted the reads using method described in Akama et al.^32^. Thus, for each gene in our polymorphism dataset, we obtained expression data that are specific to either homeolog. For the diploids, the accessions used for RNA-seq are the same genotype as the reference genomes, so no read sorting was necessary. We estimated expression levels using HTseq v0.6.1 to count reads, and then calculated reads per kilobase of transcript per million mapped reads (RPKM). The mean RPKM values from three libraries of each genotype from leaf or root tissues were used to make a distribution in RPKM that corresponds to our polymorphism gene dataset. The distribution of RPKM was used to determine the upper and lower 10% tails in expression for both homeologs separately.

### Detection of high-impact mutations

We used SnpEff v4.2^69^ to detect genetic variants that have putative loss-of function mutations in both subgenomes of *A. kamchatica*. We ran the program separately on the variant file of each subgenome. First, we built custom databases for each parental genome using our v2.2 parental assemblies and annotation. Since SnpEff ignores filter fields in VCF files, we have removed all variants that failed our filters, replaced all genotypes that failed genotype filters with no-calls (i.e.,./.), and removed any entries without valid variant calls. Such filtering allowed us to extract accurate gene summaries from SnpEff output.

SnpEff annotated polymorphisms within 32,410 and 31,119 genic regions in *A. halleri*-derived and *A. lyrata*-derived genomes, respectively. These include all mutations with any impact type, but we focused only on frameshifts, premature stop codon, loss-of-stop codons, and loss-of-start codons. The gene sets were thus reduced to 31,193 and 31,119 genes for *A. halleri-* and *A. lyrata*-derived genomes, respectively. There are 21,419 and 21,463 reciprocal best BLAST hits between *A. halleri* or *A. lyrata*, respectively, and *A. thaliana*. Based on the intersection of these two datasets, we identified 20,292 homeologs between *A. halleri* and *A. lyrata*. Out of these, 19 *halleri*-origin and 18 *lyrata-*origin genes had no coverage.

## Electronic supplementary material

Supplementary Information

## Data Availability

Illumina reads of *A. kamchatica* were submitted to DDBJ (https://www.ddbj.nig.ac.jp/dra/index-e.html) under BioProject Submission ID PRJDB6166 (see Supplementary Table 3 for all BioSample IDs). The KWS and TWN accessions analyzed by Novikova et al.^31^ were submitted to DDBJ under BioProject code PRJDB4504. Long-insert libraries used for the *A. lyrata* assembly were submitted to DDBJ under BioProject ID PRJDB6736 and BioSample ID SAMD00112182. The *A. lyrata* genome assembly is available at the European Nucleotide Archive (ENA; https://www.ebi.ac.uk/ena), under project ID PRJEB22152 and accession ID GCA_900205625. Gene alignments for homeolog-specific PCR validation, along with a summary table are available at 10.5061/dryad.15rs596. Code for *A. lyrata* genome assembly is available at https://gitlab.com/rbrisk/AlyrAssembly. Code for variant calling in *A. kamchatica* is available at https://gitlab.com/rbrisk/AkamVariants. Shell scripts for population genetics are available at https://gitlab.com/tpaape/akam_popgen_shell.
