# Supplementary material for: Patterns of polymorphism and selection in the subgenomes of the allopolyploid Arabidopsis kamchatica
Source: Nat Commun. 2018 Sep 25;9:3909. doi: 10.1038/s41467-018-06108-1 (PMC6156220; doi:10.1038/s41467-018-06108-1)
Supplement: Supplementary file 1 — Supplementary Information [file 41467_2018_6108_MOESM1_ESM.pdf]

Patterns of polymorphism and selection in the subgenomes of the allopolyploid *Arabidopsis kamchatica*

Timothy Paape, Roman V. Briskine, Gwyneth Halstead-Nussloch, Heidi E.L Lischer, Rie Shimizu-Inatsugi, Masaomi Hatakeyama, Kenta Tanaka, Tomoaki Nishiyama, Renat Sabirov, Jun Sese, Kentaro K. Shimizu

Supplementary Methods

Reference genome assembly of *A. lyrata* subsp. *petraea*

We assembled the genomes of *A. halleri* subsp. *gemmifera* (W302)[1] collected from the Tada mine in Japan and *A. lyrata* subsp. *petraea* (lyrpet4) collected from Siberia representing each of the closest known diploid parents of *A. kamchatica*[2,3]. Both *A. halleri* and *A. lyrata* are predominantly self-incompatible (SI). To reduce heterozygosity, we selfed *A. halleri* five times using bud pollination[4]. The Siberian *A. lyrata* genotype (lyrpet4) lost SI in its natural habitat, so we were able to perform two rounds of regular self-fertilization. Previously, we reported medium quality assemblies (v1.0) for both of these genotypes[5] as well as an improved version of *A. halleri*[1]*.* Here, we provide an improved version of the *A. lyrata* lyrpet4 assembly that was generated using the pipeline described by Briskine *et al*. (2016)[1] for *A. halleri* W302 and we refer to the new assemblies as version 2.2 (v2.2).

We created long-insert mate-pair libraries to complement the short-insert libraries published by Akama *et al.* (2014)[5]. We used the leaf tissue of *A. lyrata* lyrpet4 to construct the mate-pair libraries with Illumina Nextera Mate-Pair Library Prep kit modified for large insert sizes. After tagmentation with Mate Pair Tagment Enzyme, the DNA was separated by pulsed field electrophoresis into variable ranges of 22–38 kb, 15–22 kb, 11–15 kb, 7–11 kb, 5.0–7 kb, and 3.0–5.0 kb. For each range, 270–600 ng of DNA was recovered using a Zymoclean Large Fragment DNA Recovery Kit. After circularization, exonuclease treatment, fragmentation with Covaris S1, A-tailing, and adapter ligation, 14 cycles of PCR were carried out for 22–38 kb, 15–22 kb, and 11–15 kb fraction, and 10 cycles for the 7–11 kb, 5.0–7kb, and 3.0–5.0 kb fractions. After quantification of the libraries by qPCR using KAPA Library Quantification Kit for Illumina platforms, four additional cycles of PCR were performed for the 22–38 kb and 7–11 kb fractions. The libraries were purified with an AMpure XP kit, quantified with the KAPA kit again, and mixed based on the measurement. The libraries were sequenced on Illumina HiSeq 2500 at the Functional Genomics Center Zurich (http://www.fgcz.ch).

The *A. lyrata* genome was assembled from all available untrimmed read libraries with ALLPATHS-LG R50599[6] using the default parameters in two steps. In the first step, we specified expected insert sizes. In the second step, we switched to the insert sizes reported by ALLPATHS-LG in the first step. The assembly job had a peak memory utilization of 191 Gb and was completed in 84 hours on a Linux server using 30 cores.

Genome annotation of *A. lyrata* subsp. *petraea*

Both parental genomes were annotated using the same pipeline based on the recommendations from the AUGUSTUS Development Team[7]. The details for *A. halleri* can be found in Briskine *et al.* (2016)[1]. Here, we provide a brief description of the *A. lyrata* lyrpet4 annotation process (see the pipeline flowchart by Briskine *et al.* (2016)[1]. First, we aligned un-stranded paired-end 100 bp reads from *A. lyrata* W1739_L2 (leaf) and W1739_R0 (root) libraries from Paape *et al*. (2016)[8] against the *A. lyrata* lyrpet4 assembly using STAR v2.4.0i [9]. We extracted intron hints from the alignments and combined them with *nonexonpart* hints obtained from the RepeatMasker v4.0.5[10] output. The combined hints were supplied to AUGUSTUS v3.0.3 for the initial run. These obtained gene models were used to extract exon–exon junction sequences against which we aligned the original RNA-seq reads using bowtie2 v2.2.4[11]. We merged exon-exon junction alignments with the alignments to the complete reference genome and used the combined data to produce intron hints for the final AUGUSTUS run. Human readable functional descriptions were added using the AHRD tool[12]. Reciprocal best BLAST hits were calculated individually between *A. halleri* W302 or *A. lyrata* lyrpet4 and *A. thaliana* TAIR10 by aligning all coding sequences using NCBI BLAST+ v2.2.29 and comparing the scores for hits longer than 200 bp. Similarly, we calculated reciprocal best BLAST hits between W302 or lyrpet4 and *A. lyrata* subsp. *lyrata* annotation v2.0 of strain MN47 v1.07 released by Rawat *et al.* (2015)[13] for the Joint Genome Initiative (JGI) reference genome v1.07.

Improving diploid assemblies using synteny

Both *A. halleri* and *A. lyrata* diverged recently[3,14] and each has eight chromosomes[15] allowing us to use the *A. lyrata* subsp. *lyrata* strain MN47 v1.07 reference assembly[16] to perform genome-wide synteny analysis. The complete genome, coding sequences, and gene annotation of *A. lyrata* JGI were downloaded from the Phytozome v9.0 website (http://phytozome.jgi.doe.gov). Coding sequences of *A. lyrata* JGI were aligned to our *A. lyrata* lyrpet4 assembly using BLAT v3.5[17] with default parameters except maximum intron size. Because the longest intron in the *A. lyrata* lyrpet4 assembly was 44,703 bp, we set the maximum intron size to 50 kb. Hits were filtered, sorted, and merged into syntenic regions using custom Perl scripts (see the GitLab repository). We only considered the hits covering at least 85% of the query sequence and accepted the hit from a syntenic gene even when it did not have the highest score for the locus. If an *A. lyrata* lyrpet4 scaffold contained two neighboring loci that were syntenic to two *A. lyrata* JGI regions located on different chromosomes or more than 100 kb apart, the scaffold was split into two parts by removing the sequence of unknown nucleotides. Scaffolds were only split if the sequence of unknown nucleotide N's at the cut site spanned at least 50 bp. After this correction, the scaffolds were sorted by length in descending order and named sequentially beginning with scaffold_1. Because *A.*

kamchatica is a self-compatible species, we were able to remove most heterozygosity by self-fertilization and we treated both subgenomes separately as haploid (i.e. 8 homozygous chromosomes in each subgenome). Because three tandemly duplicated copies of *HMA4* were assembled on a single *A. halleri* scaffold (scaffold_0116), we compared the synteny of this region with our *A. lyrata* subsp. *petrea* assembly, *A. lyrata* JGI, and *A. thaliana* (Fig. 2A, main text), which each contain only a single *HMA4* copy. This was necessary to compare genetic diversity of homeologs between the two subgenomes of *A. kamchatica* over putatively syntenic regions (see main text Methods for details). Alignments for the 118 genes in Fig. 2 in the main document with putative roles in metal tolerance, hyperaccumulation, metal ion transport, and metal homeostasis were collected from the following resources:[18–24].

Supplementary Note 1

Reference assembly statistics

Our new *A. lyrata* assembly reduced the number of scaffolds from 281,536 from a previous version (v1.0, reported by Akama *et al.* (2014)[5] to 1,675 in version 2.2. The genome sizes of our diploid genome assemblies are 196 Mb (of which 78.9 Mb is genes) for *A. halleri* and 175 Mb (of which 75.4 Mb is genes) for *A. lyrata* (Table 1, main text). Using flow cytometry, we estimated the genome size of *A. halleri* to be 250 Mb and for *A. lyrata* it is 225 Mb, indicating that our assembled genomes captured 78% and 77% of the total genomes of both species respectively. Using flow cytometry, we estimated a genome size of 460-480 Mb for *A. kamchatica* (with some variation between genotypes), indicating that the combined genome sizes of both diploids are very close to flow cytometry estimates for the allopolyploid.

The number of annotated genes in the *A. lyrata* v2.2 assembly (31,232) is similar to the number in our *A. halleri* (Tada mine) v2.2 assembly (32,553), and to previously published *A. lyrata* subsp. *lyrata*[16] and *A. thaliana* gene annotations (Supplementary Table 1). Using reciprocal BLAST hits (RBH) to determine orthology of the annotated gene models to *A. thaliana,* we found 21,433 *A. halleri* and 21,472 *A. lyrata* genes could be assigned to a TAIR10 gene ID. Based on these results, we identified 23,529 *halleri*-origin and *lyrata*-origin homeologs (Supplementary Table 2). Our *A. halleri* and *A. lyrata* v2.2 genome assemblies also show very similar numbers of BLAST hits to the JGI *A. lyrata* genome (Supplementary Table 3).

Supplementary Note 2

Homeolog-specific PCR

We performed Sanger sequencing using homeolog-specific PCR to validate the read sorting method using *halleri*- or *lyrata*-origin SNPs for the following genes (TAIR10 IDs): AT1G02180, AT1G02290, AT1G02630 (*lyrata* only), AT1G17770, AT3G17360, AT3G10570, AT3G17611, AT4G01860 (*lyrata* only), AT4G26610, AT4G36080 (only the *halleri*-derived homeolog of KWS), AT5G13930: *CHS*, AT5G14750: *WER*. Sequence fragments ranged from 170 bp to 1,500 bp comprising a total of ca. 10 kb in length for the MUR, PAK and KWS accessions (OKH accession was used for the WER *halleri*-homeolog)[2,25]. We defined SNP positions based on differences between homeologous regions, where sequences were often enriched for SNPs due to highly divergent intron polymorphisms. Only three SNPs in Sanger sequences were different from the NGS data out of 1,375 total SNPs. However, the other SNPs in these sequences corresponded perfectly to their respective homeologous sequences and therefore still validated the read sorting method. We also had cases where double peaks were present in the Sanger sequences for one of the two homeologs, but in all cases the two SNPs corresponded to those shown in the NGS data for both homeologs, so both homeologs were partially amplified. We nevertheless consider these cases as supporting the NGS data since one homeolog was supported by Sanger data and both alleles were present in the other sequences.

Supplementary Note 3

Population structure

We used 1,000 randomly selected coding sequence (CDS) alignments from both *halleri*- and *lyrata*-derived homeologs. We then individually concatenated the *halleri* alignments and the *lyrata* alignments to use for population structure and phylogenetic analysis. The input data sets for the population structure analysis contained 21,341 and 16,223 markers from *halleri*- and *lyrata*-origin CDSs respectively. We ran STRUCTURE v2.3.4[26] ten times for each K = 1 to 9 clusters using the admixture model and 50,000 MCMC rounds for burnin followed by 100,000 rounds to generate the data. The output was analyzed with STRUCTURE HARVESTER v0.6.94 and clusters were rearranged with CLUMPP v1.1.2. (Supplementary Fig. 2).

For phylogenetic analysis for each subgenome, we added *A. halleri* or *A. lyrata* as an outgroup and ran Mr. Bayes v3.2.6[27] with default parameters for 500,000 generations sampling every 1000th generation. Phylogenetic relationships of the 25 accessions were consistent with population structure clustering described above. In each of the three phylogenies (i.e., *lyrata* subgenome, *halleri* subgenome, both homeologs combined), three clades are fairly well resolved: one large clade from the southern species range (most of Japan), another main clade from the northern range containing samples from Far East Russia and Alaska (Supplementary Fig. 3), and a separate small clade containing *A. kamchatica* subsp. *kawasakiana* accessions along with a few

divergent accessions of *A. kamchatica* subsp. *kamchatica*. However, the relationship between these clades is different between the subgenomes. The clade containing subsp. *kawasakiana* is sister to the large Japanese clade in the *lyrata*-derived subgenome and it is sister to the Russia/Alaska clade in the *halleri*-derived subgenome (Supplementary Fig. 3). Different structure assignments and phylogenetic branching patterns between the subgenomes is not compatible with the scenario of a single origin of polyploidization, and supports that multiple parental individuals contributed to the origin of *A. kamchatica*.

Supplementary Note 4

Gene ontology analysis of loss-of-function mutations

For each subgenome, we conducted gene ontology (GO) analysis to determine whether there was enrichment for GO terms using the two most common high-impact mutation types, frameshift mutations and stop codons. The H-origin gene list consisted of 3,311 copies with frameshift mutations and 1,662 genes with premature stop codons (stop gained) (Supplementary Table 10). The L-origin gene list consisted of 4,014 genes with frameshift mutations and 2002 genes with premature stop codons (stop gained). GO analysis was performed using agriGO (bioinfo.cau.edu.cn/agriGO) using a custom set of containing 19,936 GO annotations as the search background that corresponded to *A. thaliana* orthologs with reciprocal-best BLAST hits for both homeologs. The query total in Supplementary Table 11 therefore corresponds to the numbers of genes in the H-origin and L-origin list with GO annotations in our custom *A. thaliana* ortholog list. We used only queries with at least 20 genes. For the list of genes with high impact mutations in both homeologs (511 genes, Supplementary Table 10), we included the total number of genes with any mutation type. Here again, the query total in Supplementary Table 11 corresponds to the numbers of genes in the H-origin and L-origin list with GO annotations in our custom *A. thaliana* ortholog list. For both subgenomes, hydrolase activity (GO:0016787) was the most significant GO term for molecular function, followed by several GO categories for nucleotide binding (Supplementary Table 11). Programmed cell death (GO:0012501) and apoptosis (GO:0006915) were significant in the *halleri*-origin genes only.

Supplementary Figures

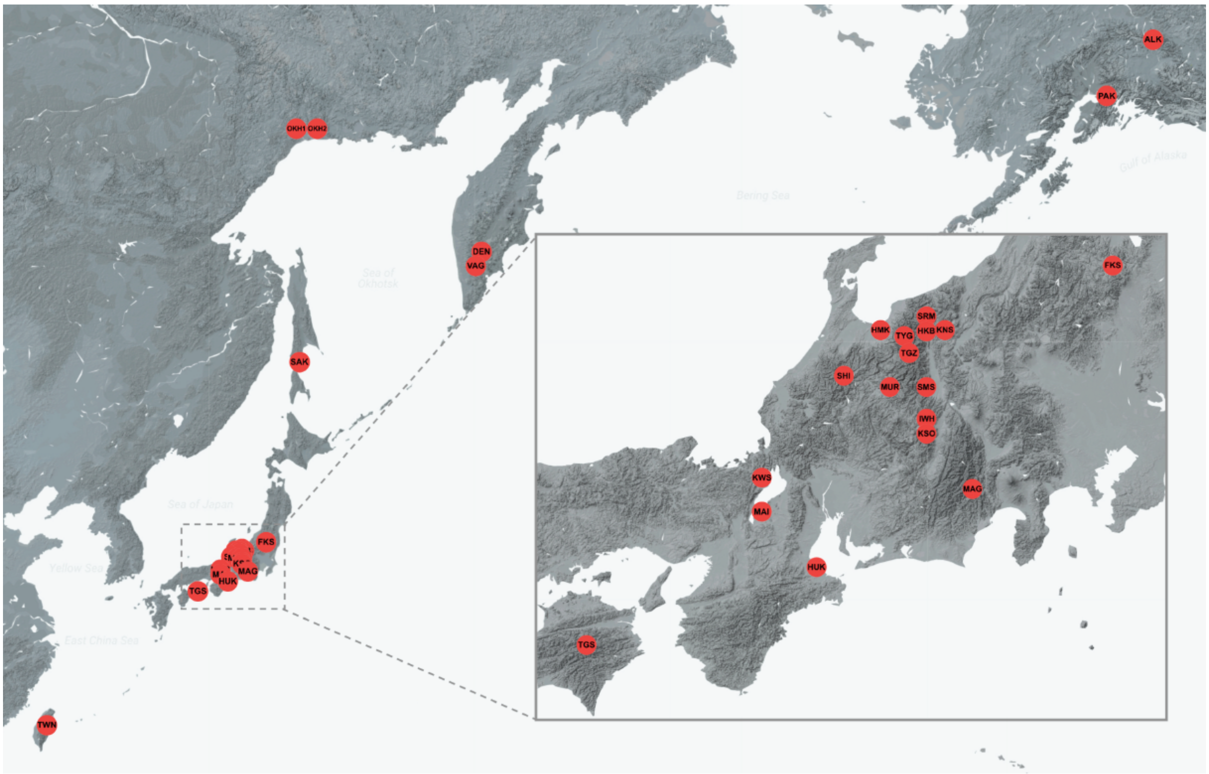

Supplementary Fig. 1. Map of 25 *Arabidopsis kamchatica* accessions sequenced in this study. Created using GPS coordinates given in Supplementary Table 3 using https://snazzymaps.com/ (all styles are licensed under creative commons and are completely free to use). Note that for populations OKH1 and OKH2 (Eastern Russia), and TGZ and TYG (Central Japan), the overlapping points in the figure have been shifted slightly for visibility.

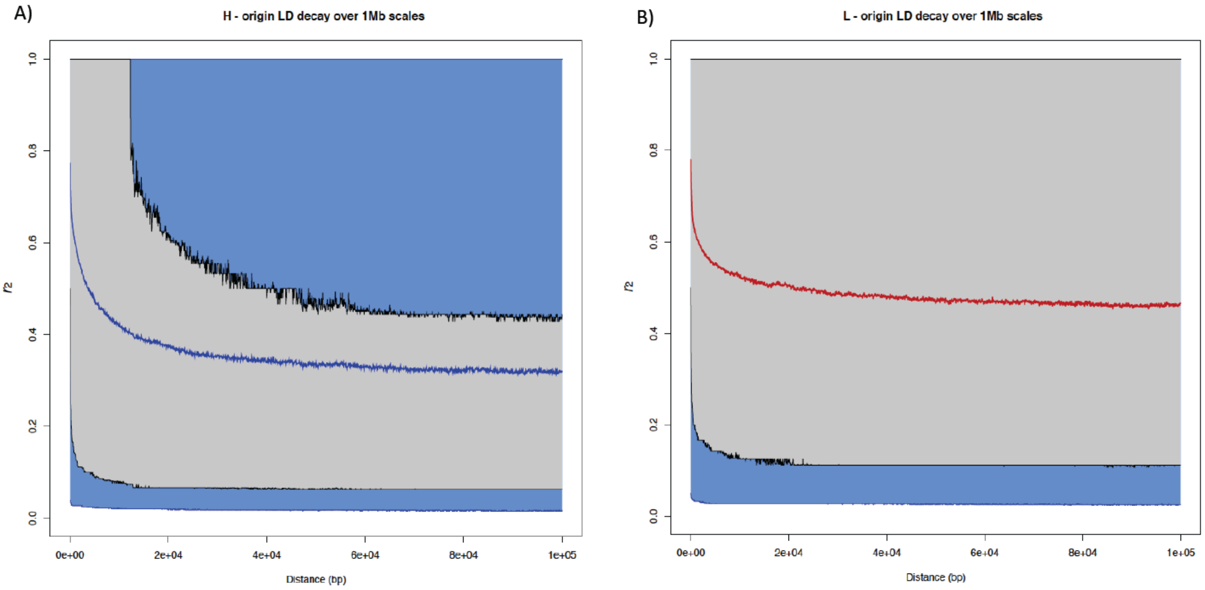

Supplementary Fig. 2. Linkage disequilibrium of *halleri*-origin (A) and *lyrata*-origin (B) subgenomes using 1 Mb windows along scaffolds. The blue (A) and red (B) curves represent the mean LD decay, while the gray region is the 50% confidence interval, and the blue region is the 90% confidence interval surrounding the means. The mean *lyrata*-origin LD remains at 0.47 while the *halleri*-origin LD levels off at 0.34 at the scale of 100 kb genomic regions.

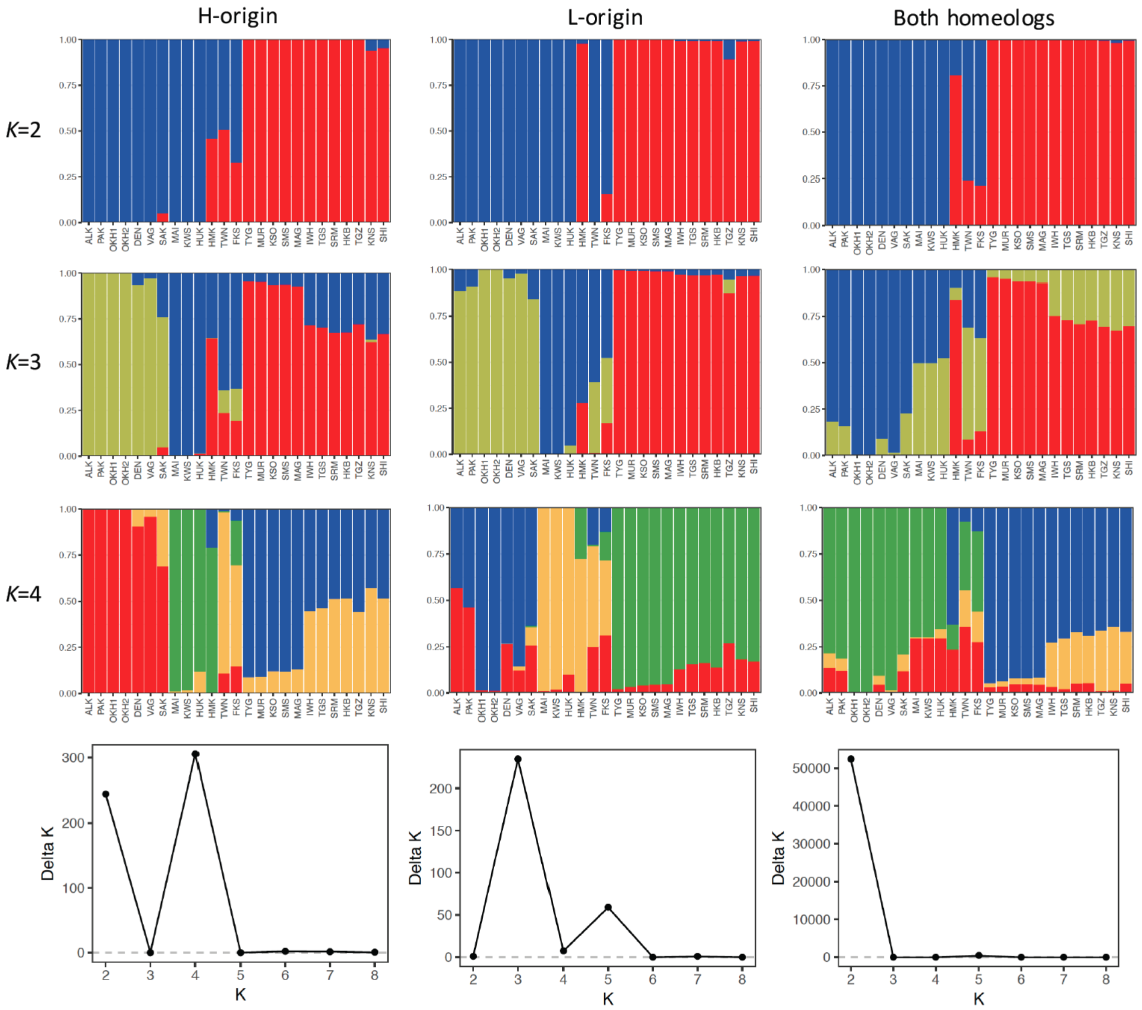

Supplementary Fig. 3. STRUCTURE assignments of *halleri* (H-origin)- and *lyrata* (L-origin)-derived homeologs for 25 *A. kamchatica* accessions for *K* = 2 to *K* = 4. The third column is the STRUCTURE assignments using SNPs from both homeologs combined. The Delta K[28] plots show the most likely *K* group clustering to be *K* = 4 for H-origin, *K* = 3 for L-origin and *K* = 2 using SNPs from both homeologs.

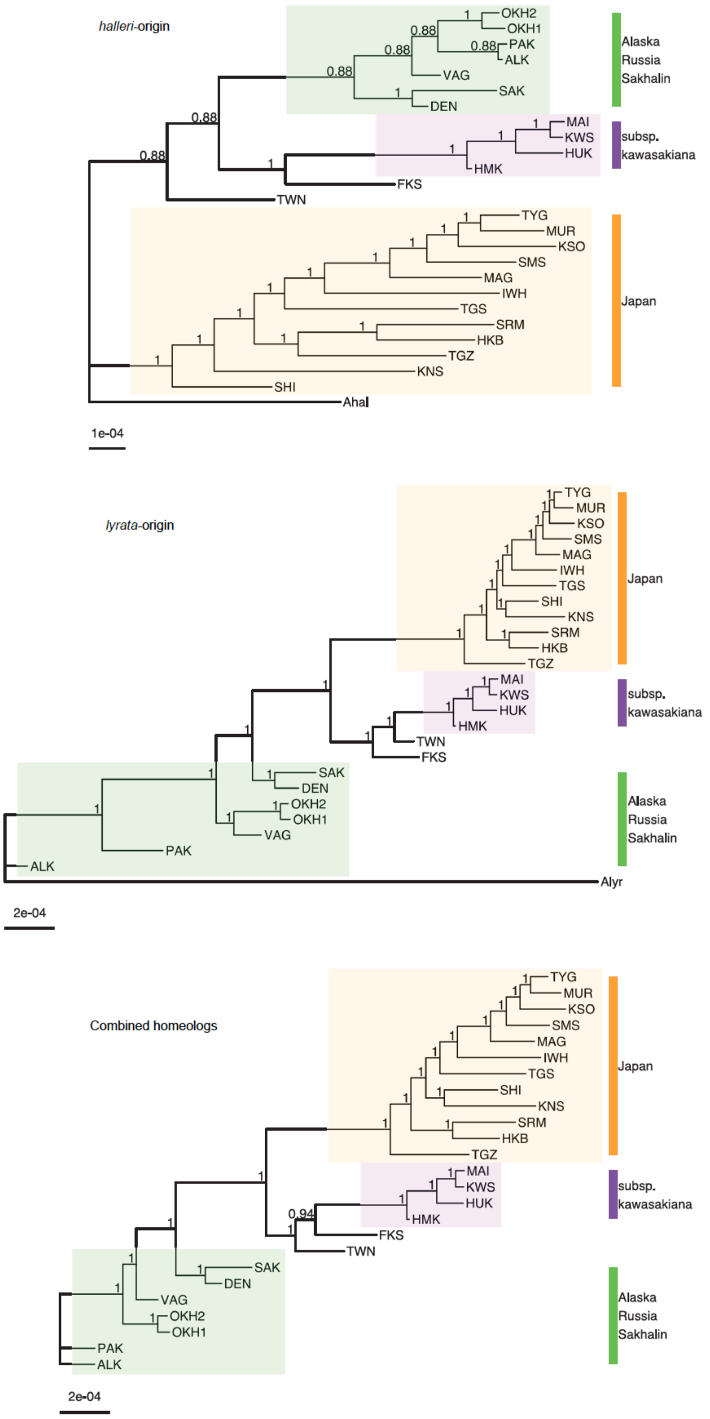

Supplementary Fig. 4. Phylogenetic relationships of 25 *A. kamchatica* accessions (top: *halleri*-subgenome; middle: *lyrata*-subgenome; bottom: both homeologs combined). Homeolog-specific trees show clustering of a large clade of Japanese accessions (orange), and a distinct clade of northern-latitude accessions (green) that are all *A. kamchatica* subsp. *kamchatica*. The small clustering of the *A. kamchatica* subsp. *kawasakiana* accessions is shown in purple, and is sister to the Japan clade in the *lyrata*-derived phylogeny, but sister to the Alaska/Russia in *halleri*-derived phylogeny. One accession from Taiwan is basal to the *kawasakiana* clade, and this lineage also contains an accession from Fukushima, Japan (FKS).

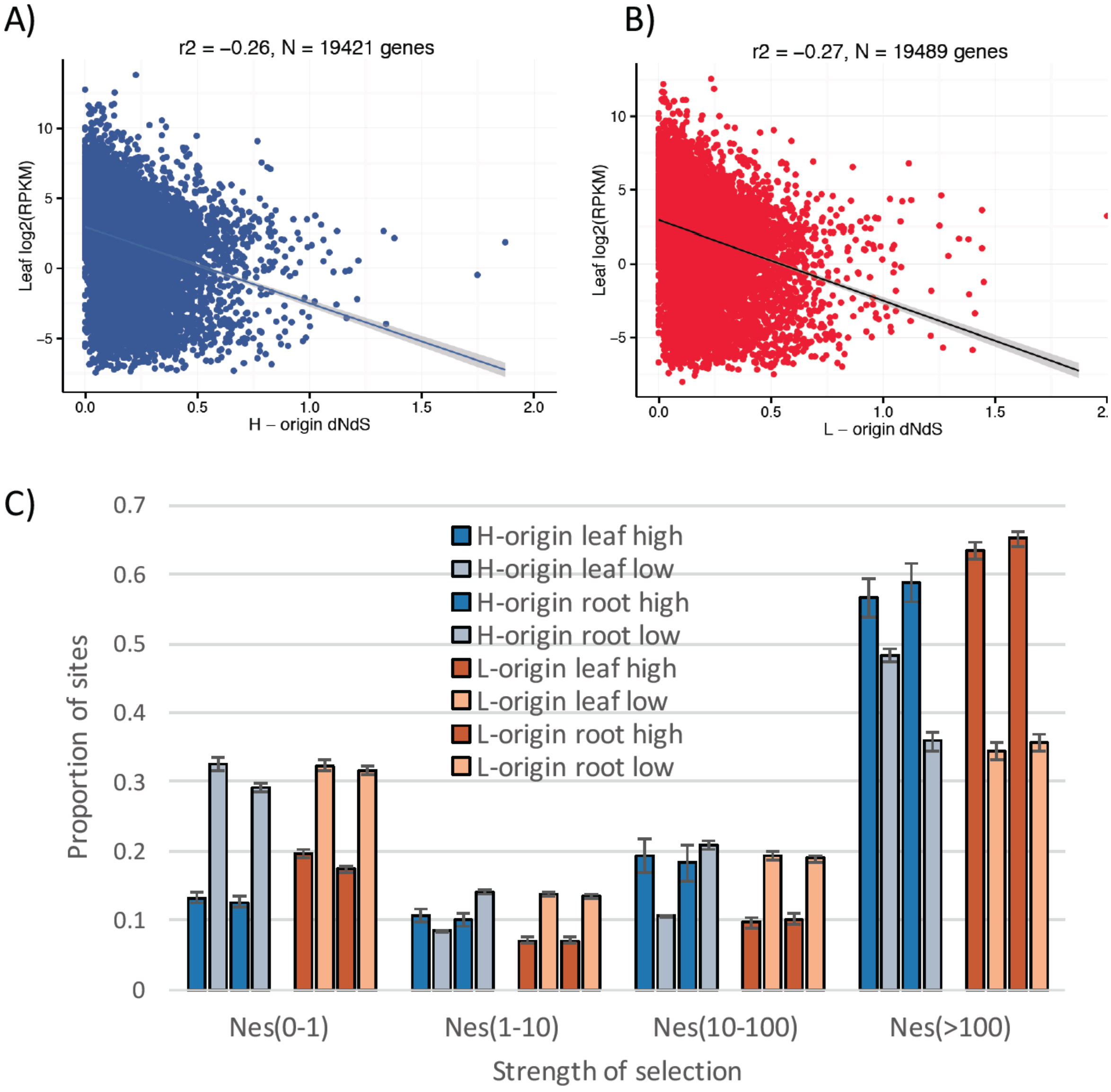

Supplementary Fig. 5. Gene expression and selective constraint. (A and B) Evolutionary rates are negatively correlated with gene expression in both homeologs. (C) DFE categorized by leaf and root expression levels in both subgenomes. Expression categories were taken from the upper 10% (high) and lower 10% (low) of expression distribution in all *A. kamchatica* homeologs.

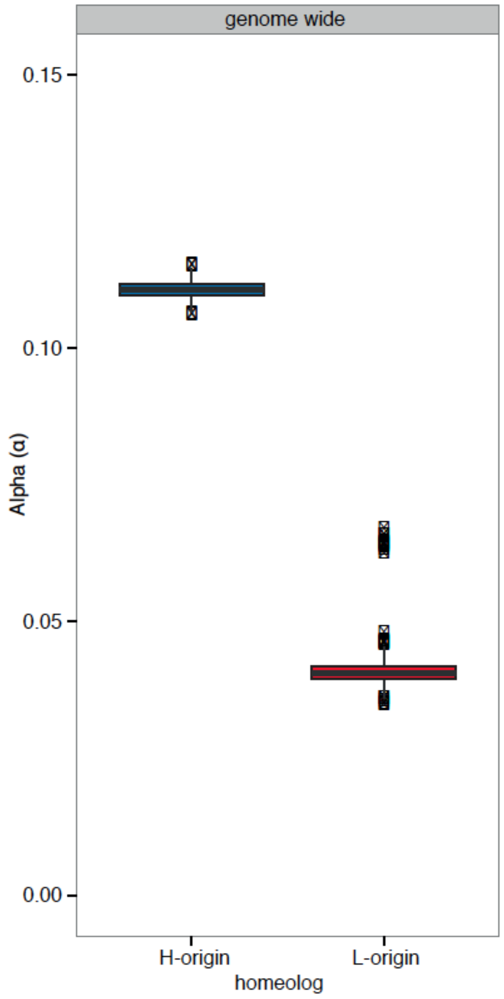

Supplementary Fig. 6. Estimates of adaptive evolution with all 25 *A. kamchatica* accessions. Mean α for H-origin was 0.11 (CI: 0.108, 0.114) and for L-origin α was 0.04 (CI: 0.037, 0.044). CI are 95% confidence intervals.

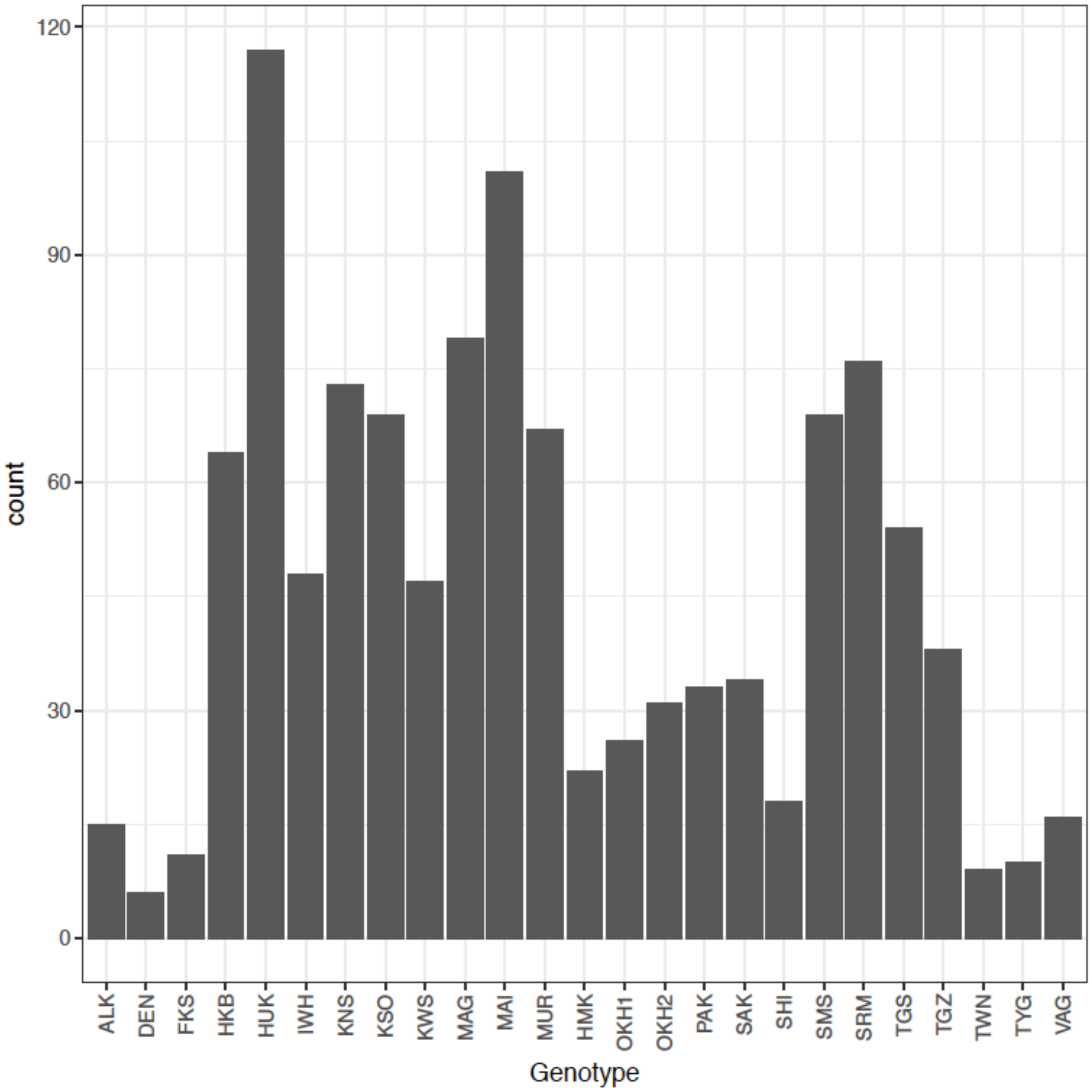

Supplementary Fig. 7. Frequencies of genes with high-impact mutations in each genotype when both homeologs have disruptive mutations (the distribution of 511 genes is from Supplementary Table 7 below).

Supplementary Tables

Supplementary Table 1. The number of genes annotated in *A. halleri* and *A. lyrata* assemblies

Annotation	Genes	mRNA	Exons
A. halleri v2.2[a]	32,553	34,553	187,838
A. lyrata v2.2[b]	31,232	33,157	181,219
A. lyrata JGI[c]	32,670	32,670	NA
A. thaliana[d]	28,775	35,386	215,909

[a] v2.2 of *A. halleri* subsp. *gemmifera* (Tada mine).

[b] v2.2 of Siberian *A. lyrata* subsp. *petraea.*

[c] Gene annotations [13] of the Joint Genome Institute (JGI*) A. lyrata* (MN47 v1.07) genome assembly[16] shown here for comparison.

[d] *A. thaliana* genome annotation from TAIR10

Supplementary Table 2. Reciprocal best BLAST hits among four genome assemblies of *Arabidopsis* species using our v.2.2 gene annotations in Supplementary Table 1[a].

Annotation A	Annotation B	Hits A on B	Hits B on A	RBH
A. halleri v2.2	*A. lyrata* v2.2	28,728	27,895	23,529
A. halleri v2.2	*A. thaliana*	25,328	23,728	21,433
A. halleri v2.2	*A. lyrata* JGI	26,402	26,917	22,447
A. lyrata v2.2	*A. lyrata* JGI	25,820	26,985	22,894
A. lyrata v2.2	*A. thaliana*	24,689	23,720	21,472
A. thaliana	*A. lyrata* JGI	24,033	25,716	21,941

[a] Only the longest transcript per gene was selected for the analysis. Hits A on B: hits from BLAST alignment of genes from the gene annotation A against the gene annotation B; RBH: reciprocal best BLAST hits. The *A. lyrata* MN47 v1.07 genome assembly by Hu *et al.*[16] is available from JGI and annotation from Rawat *et al.*[13]. The *A. thaliana* annotation is available at TAIR (https://www.arabidopsis.org/).

Supplementary Table 3. List of 25 *A. kamchatica* accessions, sampling locations and sequencing depth [a]

Accession	Species	location	lat_lon	Reads	Total_Coverage	Sorted_Ahal	Sorted_Alyr	Sorted_Common	Total_Ahal	Total_Alyr	Cov_Ahal	Cov_Alyr	SNPs_Ahal	SNPs_Alyr	BioSample
ALK	Arabidopsis kamchatica subsp. kamchatica	USA: Alaska, Richardson Highway, South of Darling Creek bridge	63.4N 145.8W	41,222,868	8.8	14,118,890	10,796,478	1,121,305	15,172,336	11,851,960	7.8	6.8	170,099	267,382	SAMD00089941
DEN	Arabidopsis kamchatica subsp. kamchatica	Russia: Kamchatskii krai, near the river Denokhonok	54.2N 158.1E	29,858,928	6.3	10,393,815	7,703,567	764,879	11,122,492	8,427,447	5.7	4.9	109,229	211,658	SAMD00089945
FKS	Arabidopsis kamchatica subsp. kamchatica	Japan: Fukushima	37.2N 139.9E	40,709,648	8.7	14,460,719	10,002,155	1,729,908	16,138,372	11,676,183	8.3	6.7	167,396	320,181	SAMD00089940
HKB	Arabidopsis kamchatica subsp. kamchatica	Japan: Hakubayari	36.7N 137.8E	83,323,614	17.7	29,795,327	22,072,831	2,089,056	31,789,560	24,049,797	16.4	13.9	270,403	553,738	SAMD00089930
HMK	Arabidopsis kamchatica subsp. kawasakiana	Japan: Toyama	36.7N 137.3E	70,437,142	15	23,995,643	17,543,808	1,878,282	25,794,172	19,323,093	13.3	11.1	279,514	496,611	SAMD00089926
HUK	Arabidopsis kamchatica subsp. kawasakiana	Japan: Hukiinoura	34.6N 136.6E	106,898,796	22.7	37,136,448	27,677,971	2,750,024	39,756,647	30,274,808	20.5	17.5	340,598	617,775	SAMD00089924
IWH	Arabidopsis kamchatica subsp. kamchatica	Japan: Iwahana	35.9N 137.8E	52,549,196	11.2	18,872,866	14,148,184	1,425,613	20,239,313	15,500,712	10.4	8.9	229,511	476,579	SAMD00089935
KNS	Arabidopsis kamchatica subsp. kamchatica	Japan: Kinasa	36.7N 138.0E	91,705,866	19.5	32,746,897	24,388,091	2,336,948	34,975,970	26,595,190	18	15.3	289,477	583,765	SAMD00089928
KSO	Arabidopsis kamchatica subsp. kamchatica	Japan: Kisokomagatake	35.8N 137.8E	82,333,488	17.5	29,916,953	22,331,282	2,216,628	32,037,117	24,432,965	16.5	14.1	273,080	553,639	SAMD00089929
KWS	Arabidopsis kamchatica subsp. kawasakiana	Japan: Takashima	35.4N 136.0E	57,556,260	12.2	18,879,180	13,938,194	1,710,830	20,528,832	15,573,108	10.6	9	214,919	385,250	SAMD00045705
MAG	Arabidopsis kamchatica subsp. kamchatica	Japan: Magosazima	35.3N 138.3E	100,480,490	21.4	35,909,855	26,847,065	2,604,139	38,400,425	29,310,981	19.8	16.9	290,361	593,827	SAMD00089925
MAI	Arabidopsis kamchatica subsp. kawasakiana	Japan: Maiamihama	35.1N 136.0E	99,558,822	21.2	34,004,362	25,237,282	2,475,873	36,363,640	27,575,460	18.7	15.9	324,490	585,919	SAMD00089927
MUR	Arabidopsis kamchatica subsp. kamchatica	Japan: Murodo	36.2N 137.4E	92,156,804	19.6	28,450,072	20,969,486	1,884,357	30,239,914	22,723,204	15.6	13.1	259,583	519,488	SAMD00089932
OKH1	Arabidopsis kamchatica subsp. kamchatica	Russia: Khabarovsk krai, Okhotskii raion, foothills of Mt. Lanzhinskie gory	59.4N 143.3E	47,972,636	10.2	16,240,333	12,120,236	1,057,909	17,225,075	13,098,741	8.9	7.6	208,143	400,845	SAMD00089938
OKH2	Arabidopsis kamchatica subsp. kamchatica	Russia: Khabarovsk krai, Okhotskii raion, foothills of Mt. Lanzhinskie gory	59.4N 143.3E	45,097,050	9.6	15,717,431	11,766,389	1,179,854	16,842,632	12,882,931	8.7	7.4	197,546	380,656	SAMD00089939
PAK	Arabidopsis kamchatica subsp. kamchatica	USA: Alaska, Potter	61.2N 149.3W	180,109,152	38.3	27,117,743	20,836,034	2,642,737	29,631,690	23,318,884	15.3	13.4	214,144	361,332	SAMD00089933
SAK	Arabidopsis kamchatica subsp. kamchatica	Russia: Sakhalin, Marakovskii raion, Zaozernoye	48.4N 142.7E	51,986,358	11.1	16,978,097	12,521,408	1,214,392	18,133,252	13,663,380	9.3	7.9	208,332	408,064	SAMD00089937
SHI	Arabidopsis kamchatica subsp. kamchatica	Japan: Shirakawa	36.3N 136.9E	36,818,198	7.8	12,491,175	9,099,814	863,158	13,310,606	9,909,963	6.9	5.7	146,395	294,813	SAMD00089943
SMS	Arabidopsis kamchatica subsp. kamchatica	Japan: Simasimadani	36.2N 137.8E	82,024,638	17.4	28,623,779	21,103,492	2,121,047	30,651,632	23,110,874	15.8	13.3	267,098	535,000	SAMD00089931
SRM	Arabidopsis kamchatica subsp. kamchatica	Japan: Mt. Shirouma	36.8N 137.8E	103,381,614	22	37,373,380	27,889,832	2,881,603	40,139,114	30,636,345	20.7	17.7	295,244	601,727	SAMD00089923
TGS	Arabidopsis kamchatica subsp. kamchatica	Japan: Mt. Shikokutsurugi	33.9N 134.1E	64,747,706	13.8	23,060,935	16,670,868	1,747,686	24,729,621	18,328,385	12.7	10.6	240,958	500,049	SAMD00089934
TGZ	Arabidopsis kamchatica subsp. kamchatica	Japan: Tsurugigozen	36.6N 137.6E	49,130,130	10.4	17,080,259	12,600,990	1,278,910	18,304,390	13,814,192	9.4	8	205,685	421,797	SAMD00089936
TWN	Arabidopsis kamchatica subsp. kamchatica	Taiwan: Taroko national park	24.0N 121.3E	27,214,840	5.8	9,374,591	6,723,726	725,941	10,068,892	7,412,536	5.2	4.3	99,741	178,623	SAMD00045707
TYG	Arabidopsis kamchatica subsp. kamchatica	Japan: Tateyamagawa	36.6N 137.6E	33,247,108	7.1	11,084,924	8,145,924	755,793	11,802,582	8,856,608	6.1	5.1	127,526	255,460	SAMD00089944
VAG	Arabidopsis kamchatica subsp. kamchatica	Russia: Kamchatskii krai, near the river Vaktan GanalÕskii	53.5N 157.6E	40,538,374	8.6	13,933,714	10,460,424	960,743	14,843,746	11,362,106	7.6	6.6	171,068	331,211	SAMD00089942

[a] The samples are in the DDBJ short read archive BioProjects PRJDB6166 and PRJDB4054 (KWS and TWN only).

Supplementary Table 4. Polymorphism and nucleotide diversity statistics of both subgenomes by sliding window analysis.

H-origin	bases[a]	bases (%)[a]	polym[b]	θ_w[c]	π[d]
overall	163517656	1	1138032	0.0018	0.0017
gene	75291060	0.4604	454338	0.0016	0.0015
coding	38896876	0.2379	216194	0.0015	0.0014
intron	22946734	0.1403	154633	0.0017	0.0017
intergenic	83592223	0.5112	660511	0.0035	0.0033
L-origin	bases	Bases (%)	polym	θ_w	π
overall	149864674	1	946600	0.0017	0.0017
gene	72299008	0.4824	436107	0.0016	0.0016
coding	37093072	0.2475	205023	0.0015	0.0015
intron	21685851	0.1447	146380	0.0018	0.0018
intergenic	74042836	0.4941	496233	0.0034	0.0034

a: total number of nucleotides in each category and the proportion to the overall bases

b: polym = number of polymorphic sites in each category

c: Watterson's polymorphism estimator, θ_w

d: nucleotide diversity, π

Supplementary Table 5. Samples used for estimating nucleotide diversity, site frequency spectra and DFE and α in Fig. 4 in main text [a].

A. kamchatica	*A. lyrata*	*A. halleri*
ALK	SRR2040790_1	SRR2040780_1
DEN	SRR2040791_2	SRR2040780_2
HKB	SRR2040792_1	SRR2040782_1
IWH	SRR2040793_2	SRR2040782_2
KNS	SRR2040794_1	SRR2040783_1
KSO	SRR2040795_1	SRR2040783_2
MAG	SRR2040795_2	SRR2040784_1
MUR	SRR2040796_2	SRR2040784_2
OKH1	SRR2040797_2	SRR2040785_1
OKH2	SRR2040798_1	SRR2040785_2
PAK	SRR3111438_2	SRR2040786_1
SAK	SRR3111439_1	SRR2040786_2
SHI	SRR3111439_2	SRR2040787_1
SMS	SRR3111440_1	SRR2040787_2
SRM	SRR3111441_1	SRR2040810_1
TGS	SRR3111441_2	SRR2040810_2
TGZ	SRR3111442_2	SRR3107262_1
VAG	SRR3111443_1	SRR3107262_2

[a] Illumina reads from European *A. halleri* and *A. lyrata* were obtained from Novikova *et al.*[29]. SNPs in diploid parents were phased and separated into two alleles, indicated by _1 and _2 following accession number. To get equal sample size, *A. lyrata* alleles samples were chosen at random.

Supplementary Table 6. Nucleotide diversity (π) and Tajima's *D* estimates from *A. halleri* and *A. lyrata.*

	A. halleri					*A. lyrata*				
	Mean	Median	St. Dev	N		Mean	Median	St. Dev	N	*r*
π_{total}	0.0097	0.0077	0.0076	19693		0.0099	0.0079	0.0077	18276	0.55
π_{nonsyn}	0.0054	0.0035	0.0101	19693		0.0053	0.0035	0.0077	18396	0.48
π_{syn}	0.0281	0.0223	0.0377	19693		0.0282	0.0226	0.0255	18396	0.43
Taj *D*	-0.24	-0.26	0.78	19644		-0.39	-0.41	0.74	18254	0.09

The mean, median and standard deviation (St. Dev) around the mean are reported for N numbers of homoeologs for each test statistic. The Pearson's correlation coefficient is denoted by *r* is the correlation between both homeologs for each statistic. All p-values for correlations are < 0.0001.

Supplementary Table 7. Estimated effective population sizes (N_e) using empirical nucleotide diversity estimates and published mutation accumulation rates[a].

Species/subgenome	π_{syn}	π_{total}	N_e [b]	N_e [c]
A. kamchatica	0.0046	0.0015	77000	53571
H-origin	0.0044	0.0015	73333	52143
L-origin	0.0049	0.0015	81667	53929
A. halleri	0.028	0.0097	466667	364202
A. lyrata	0.029	0.0102	483333	345041

[a] The calculation of N_e was conducted using the equation $\pi_{syn}/4\mu$. The mutation rates μ were published by Koch *et al.*[30] who used only synonymous nucleotide diversity, and Ossowski *et al.*[31] who used total nucleotide diversity.

[b] Calculated using the mutation rate from Koch *et al.*[30] : $\mu = 1.50E^{-08}$

[c] Calculated using the mutation rate from Ossowski *et al.*[31] : $\mu = 7.00E^{-09}$

Supplementary Table 8. The number of intergenic sites used to construct two-dimensional joint site frequency spectra.

A. halleri - H-origin	SNPs[a]	*A. lyrata* - L-origin	SNPs[a]
non polymorphic	119252	non polymorphic	183452
private SNPs *A. halleri*	249368	private SNPs *A. lyrata*	221589
private SNPs H-origin	52403	private SNPs L-origin	53426
shared SNPs	89626	shared SNPs	48822
total	510649	total	507289

[a] SNPs used for the demographic analysis using the software fastsimcoal 2.6 [32] .

Supplementary Table 9. Parameter estimates of two demographic models.

A. halleri- H-origin	N_e Diploid	N_e Subgenome	N_e ANC	*Tdiv*	R0	R1	MaxEstLhood	df
M1 simple	386838	84587	409504	101151	-	-	-1250310	4
2.5%	372371	74300	383996	87527				
97.5%	407645	88101	436662	105547				
M2 exp growth	317586	83939	317940	74579	-1.3E-07	-4.0E-06	-1284541	6
2.5%	307220	77039	314511	64478	-7.8E-07	-1.6E-05		
97.5%	341770	103347	369935	79773	-1.2E-07	-2.3E-06		

A. lyrata- L-origin	N_e Diploid	N_e Subgenome	N_e ANC	*Tdiv*	R0	R1	MaxEstLhood	
M1 simple	328403	90324	345220	136871			-1020366	4
2.5%	314271	80856	325238	121550				
97.5%	347247	94448	382771	145647				
M2 exp growth	341684	88062	348720	89409	-1.2E-07	-7.0E-06	-1023247	6
2.5%	333318	74215	341263	76979	-1.1E-06	-1.5E-05		
97.5%	371305	99481	398762	99232	-1.2E-07	-2.4E-06		

[a] Model M1 estimated divergence using a stepwise model of population size change, and model M2 estimated exponential population size changes in the polyploid and diploids using the software fastsimcoal 2.6 [32]. A minimum of 100,000 and maximum of 250,000 coalescent simulations with 10-40 cycles likelihood maximization was used to estimate parameters and model likelihoods. 95% confidence intervals (in gray; lower: 2.5% and upper: 97.5%) were estimated using 100 simulated joints site frequency spectra for each of the two subgenomes and running them using the same model priors and input parameters as the empirical datasets. For both diploid-subgenome comparisons, the M1 model had significantly higher likelihoods. Parameters: N_e = effective population size, N_e ANC = ancestral effective population size, *Tdiv* = time of divergence, R0 = rate of exponential population growth of diploids, R1 = rate of exponential population growth of polyploid subgenomes.

Supplementary Table 10. High-impact mutations[a].

Homeolog	frameshift variant	start lost	stop gained	stop lost	total[b]	% total
H-origin	3311	282	1662	190	4219	20.78
L-origin	4014	423	2002	251	4952	24.39
Shared in both homeologs[c]					1559	7.68
Shared in genotypes[d]					511	2.52

[a] Counts are the number of homeologs with one or more of any of the mutation types.
[b] total number of homeologs with one or more high-impact mutations (multiple mutation types are possible in a single homeolog).
[c] total number of genes with high-impact mutations in both homeologs out of 25 individuals
[d] total number of high-impact mutations in both homeologs in an individual.

Supplementary Table 11. Gene Ontology of high-impact mutations [a].

H-origin								
GO_acc	term_type	Term	queryitem	querytotal	refitem	reftotal	pvalue	FDR
GO:0003824	F	catalytic activity	1507	4273	6350	19936	6.90E-05	0.036
GO:0016787	F	hydrolase activity	567	4273	2285	19936	0.00014	0.036
GO:0001883	F	purine nucleoside binding	260	4273	983	19936	0.00015	0.036
GO:0001882	F	nucleoside binding	260	4273	983	19936	0.00015	0.036
GO:0030554	F	adenyl nucleotide binding	260	4273	983	19936	0.00015	0.036
GO:0019825	F	oxygen binding	58	4273	159	19936	1.10E-05	0.011
GO:0012501	P	programmed cell death	45	4273	111	19936	4.60E-06	0.012
GO:0008236	F	serine-type peptidase activity	42	4273	115	19936	0.00016	0.036
GO:0017171	F	serine hydrolase activity	42	4273	115	19936	0.00016	0.036
GO:0006915	P	apoptosis	32	4273	61	19936	1.10E-07	0.00056
GO:0004888	F	transmembrane receptor activity	31	4273	64	19936	1.60E-06	0.0033
L-origin								
GO_acc	term_type	Term	queryitem	querytotal	refitem	reftotal	pvalue	FDR
GO:0016787	F	hydrolase activity	663	5031	2285	19936	5.90E-05	0.022
GO:0017076	F	purine nucleotide binding	346	5031	1146	19936	0.00013	0.03
GO:0001882	F	nucleoside binding	314	5031	983	19936	2.50E-06	0.0019
GO:0001883	F	purine nucleoside binding	314	5031	983	19936	2.50E-06	0.0019
GO:0030554	F	adenyl nucleotide binding	314	5031	983	19936	2.50E-06	0.0019
GO:0032559	F	adenyl ribonucleotide binding	294	5031	927	19936	8.80E-06	0.0044
GO:0005524	F	ATP binding	292	5031	921	19936	9.70E-06	0.0044
GO:0017111	F	nucleoside-triphosphatase activity	185	5031	574	19936	0.00013	0.03
GO:0019825	F	oxygen binding	61	5031	159	19936	0.00019	0.04
GO:0008236	F	serine-type peptidase activity	48	5031	115	19936	8.40E-05	0.024
GO:0017171	F	serine hydrolase activity	48	5031	115	19936	8.40E-05	0.024
Shared in both homeologs in a single genotype								
GO_acc	term_type	Term	queryitem	querytotal	refitem	reftotal	pvalue	FDR
GO:0060089	F	molecular transducer activity	17	497	239	19936	0.00014	0.013
GO:0004871	F	signal transducer activity	17	497	239	19936	0.00014	0.013
GO:0004872	F	receptor activity	11	497	95	19936	2.90E-05	0.0052
GO:0012501	P	programmed cell death	11	497	111	19936	0.00012	0.042
GO:0004888	F	transmembrane receptor activity	10	497	64	19936	4.50E-06	0.0016
GO:0006915	P	apoptosis	9	497	61	19936	2.10E-05	0.015

[a] GO analysis was conducted for premature stop codon and frameshift combined only for H-origin and L-origin derived coding sequences. GO analysis was also done for genes with any of the four high-impact mutation types (from Supplementary Table 10) where both homeologs in a single genotype had disruptive mutations (shared in both homeologs in a single genotype).

Supplementary References

1. Briskine, R. V. *et al.* Genome assembly and annotation of *Arabidopsis halleri* , a model for heavy metal hyperaccumulation and evolutionary ecology. *Mol. Ecol. Resour.* **5**, 1025-1036 (2016).
2. Shimizu-Inatsugi, R. *et al.* The allopolyploid *Arabidopsis kamchatica* originated from multiple individuals of *Arabidopsis lyrata* and *Arabidopsis halleri*. *Mol. Ecol.* **18,** 4024–4048 (2009).
3. Schmickl, R., Jørgensen, M. H., Brysting, A. K. & Koch, M. A. The evolutionary history of the Arabidopsis lyrata complex: a hybrid in the amphi-Beringian area closes a large distribution gap and builds up a genetic barrier. *BMC Evol. Biol.* **10,** 98 (2010).
4. Tsuchimatsu, T. *et al.* Evolution of self-compatibility in Arabidopsis by a mutation in the male specificity gene. *Nature* **464,** 1342–1346 (2010).
5. Akama, S., Shimizu-Inatsugi, R., Shimizu, K. K. & Sese, J. Genome-wide quantification of homeolog expression ratio revealed nonstochastic gene regulation in synthetic allopolyploid Arabidopsis. *Nucleic Acids Res.* **42,** e46–e46 (2014).
6. Butler, J. *et al.* ALLPATHS: De novo assembly of whole-genome shotgun microreads. *Genome Res.* **18,** 810–820 (2008).
7. AUGUSTUS Development Team. Incorporating RNAseq data into AUGUSTUS with TopHat. (2014). Available at: http://bioinf.uni-greifswald.de/bioinf/wiki/pmwiki.php?n=IncorporatingRNAseq.Tophat. (Accessed: 15th January 2015)
8. Paape, T. *et al.* Conserved but attenuated parental gene expression in allopolyploids: Constitutive zinc hyperaccumulation in the allotetraploid *Arabidopsis kamchatica*. *Mol. Biol. Evol.* **33,** 2781–2800 (2016).
9. Dobin, A. *et al.* STAR: ultrafast universal RNA-seq aligner. *Bioinformatics* **29,** 15–21 (2013).

10. Smit, A., Hubley, R. & Green, P. RepeatMasker Open-3.0. (1996). Available at: http://www.repeatmasker.org.
11. Langmead, B. & Salzberg, S. L. Fast gapped-read alignment with Bowtie 2. *Nat. Methods* **9,** 357–359 (2012).
12. Tomato Genome Consortium. The tomato genome sequence provides insights into fleshy fruit evolution. *Nature* **485,** 635–641 (2012).
13. Rawat, V. *et al.* Improving the Annotation of Arabidopsis lyrata Using RNA-Seq Data. *PLOS ONE* **10,** e0137391 (2015).
14. Roux, C. *et al.* Does speciation between *Arabidopsis halleri* and *Arabidopsis lyrata* coincide with major changes in a molecular target of adaptation? *PLoS ONE* **6,** e26872 (2011).
15. Al-Shehbaz, I. A. & O'Kane, S. L. Taxonomy and Phylogeny of Arabidopsis (Brassicaceae). *Arab. Book* **1,** e0001 (2002).
16. Hu, T. T. *et al.* The Arabidopsis lyrata genome sequence and the basis of rapid genome size change. *Nat. Genet.* **43,** 476–481 (2011).
17. Kent, W. J. BLAT—The BLAST-Like Alignment Tool. *Genome Res.* **12,** 656–664 (2002).
18. Arrivault, S., Senger, T. & Krämer, U. The Arabidopsis metal tolerance protein AtMTP3 maintains metal homeostasis by mediating Zn exclusion from the shoot under Fe deficiency and Zn oversupply. *Plant J.* **46,** 861–879 (2006).
19. Filatov, V. *et al.* Comparison of gene expression in segregating families identifies genes and genomic regions involved in a novel adaptation, zinc hyperaccumulation: GENE EXPRESSION IN SEGREGATING FAMILIES. *Mol. Ecol.* **15,** 3045–3059 (2006).
20. Talke, I. N. Zinc-dependent global transcriptional control, transcriptional deregulation, and higher gene copy number for genes in metal homeostasis of the hyperaccumulator *Arabidopsis halleri. PLANT Physiol.* **142,** 148–167 (2006).
21. Hanikenne, M. *et al.* Evolution of metal hyperaccumulation required cis-regulatory changes and triplication of HMA4. *Nature* **453,** 391–395 (2008).

22. Shahzad, Z. *et al.* The Five AhMTP1 Zinc Transporters Undergo Different Evolutionary Fates towards Adaptive Evolution to Zinc Tolerance in Arabidopsis halleri. *PLoS Genet.* **6,** e1000911 (2010).

23. Lamesch, P. *et al.* The Arabidopsis Information Resource (TAIR): improved gene annotation and new tools. *Nucleic Acids Res.* **40,** D1202–D1210 (2012).

24. Shahzad, Z. *et al. Plant Defensin type 1* (*PDF1*): protein promiscuity and expression variation within the *Arabidopsis* genus shed light on zinc tolerance acquisition in *Arabidopsis halleri*. *New Phytol.* **200,** 820–833 (2013).

25. Tsuchimatsu, T., Kaiser, P., Yew, C.-L., Bachelier, J. B. & Shimizu, K. K. Recent Loss of Self-Incompatibility by Degradation of the Male Component in Allotetraploid Arabidopsis kamchatica. *PLoS Genet.* **8,** e1002838 (2012).

26. Pritchard, J. K., Stephens, M. & Donnelly, P. Inference of population structure using multilocus genotype data. *Genetics* **155,** 945–959 (2000).

27. Ronquist, F. *et al.* MrBayes 3.2: Efficient Bayesian Phylogenetic Inference and Model Choice Across a Large Model Space. *Syst. Biol.* **61,** 539–542 (2012).

28. Evanno, G., Regnaut, S. & Goudet, J. Detecting the number of clusters of individuals using the software structure: a simulation study. *Mol. Ecol.* **14,** 2611–2620 (2005).

29. Novikova, P. Y. *et al.* Sequencing of the genus Arabidopsis identifies a complex history of nonbifurcating speciation and abundant trans-specific polymorphism. *Nat. Genet.* **48,** 1077–1082 (2016).

30. Koch, M. A., Haubold, B. & Mitchell-Olds, T. Comparative evolutionary analysis of Chalcone Synthase and Alcohol Dehydrogenase loci in *Arabidopsis, Arabis*, and related genera (Brassicaceae)). *Mol. Biol. Evol.* **17,** 1483–1498 (2000).

31. Ossowski, S. *et al.* The rate and molecular spectrum of spontaneous mutations in *Arabidopsis thaliana*. *Science* **327,** 92–94 (2010).

32. Excoffier, L., Dupanloup, I., Huerta-Sánchez, E., Sousa, V. C. & Foll, M. Robust demographic inference from genomic and SNP data. *PLoS Genet.* **9,** e1003905 (2013).
